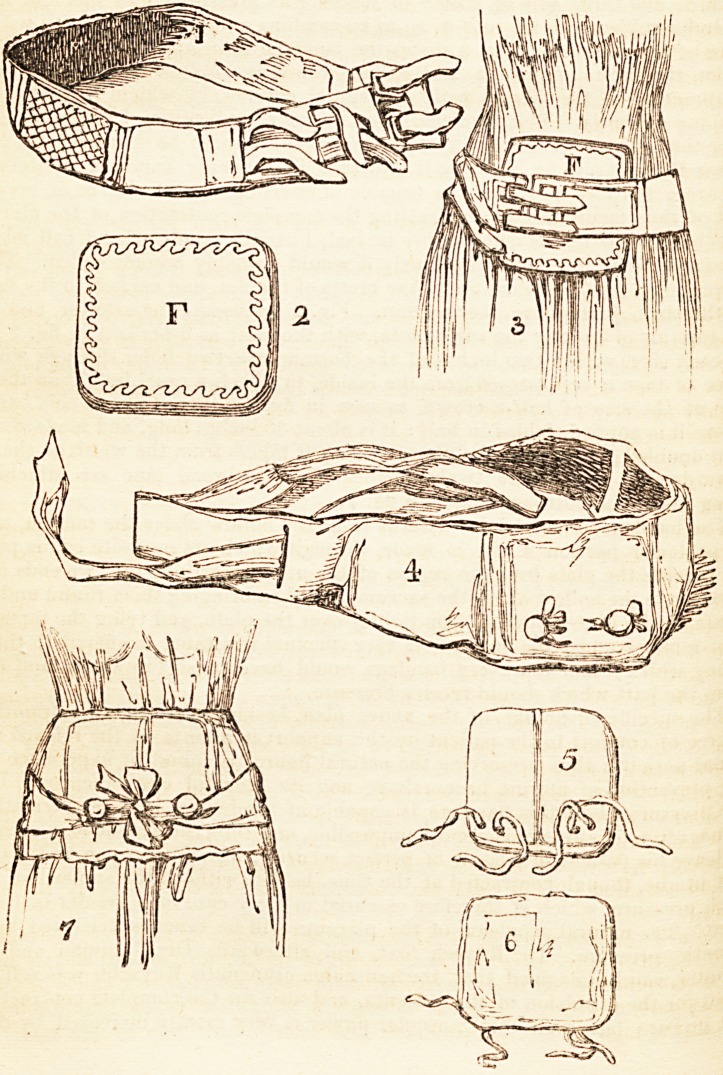# Extra-Limites

**Published:** 1839-01-01

**Authors:** 


### EXTRA-LIMITES.

### An Account of an Epidemic Cholera, which prevailed in the District of Berehaven, in Ireland, in the Autumn of 1837. By *Edmund Sharkey*, M.B. T.C.D.

During the months of September and October, 1837, an epidemic diarrhœa prevailed here to a very great extent, in very many cases combined with vomiting, and amounting to a bilious cholera; but never presenting any approach to the malignant type. It affected adults exclusively, and yielded almost universally to laudanum, given in doses of 30—40—50 drops in a little peppermint water. Very rarely it lapsed into dysentery, and required the employment of calomel, opium, and hippo, followed by astringents. About the end of October, it had nearly subsided, but at the beginning of November the malignant form of the disease appeared at Castletown; no case existing of it at the time in any part of the surrounding country. The prominent symptoms of the disease being the same as elsewhere observed, are already sufficiently known. There was one, however, which presented itself very frequently, viz. a sense of "blowing" in the ears, which I had not previously noticed. A remarkable circumstance was, the almost constant uniformity of the hour at which the disease made its attack: this was, in nine cases out of ten, a little before day-break. Whether this was merely a coincidence, or whether it depended on any particular stage of the digestive process, I will not pretend to decide; we know that the attacks of several diseases are periodical.

With regard to its mode of propagation, that is a point on which I will not now touch; I will confine myself at present to practical considerations.

The number of cases which occurred in this district, comprising the Barony of Bere, and containing a population of 15,000, was 360, out of which there were 162 deaths. The duty was divided between my friend Dr. Philip, A. Armstrong, and myself; and I believe I may safely assert that never did duty more laborious fall to the lot of medical men. Ah! little do city practitioners, who have only to go round their hospitals and issue their orders, know the difficulties, obstacles, and discouragements, which the provincial physician has to encounter in such a country as this. Judge what sort of co-operation we could expect in the treatment of such a disease as cholera, in cabins wholly destitute of all the appendages of comfort, nay even of the most indispensable household utensils, and where the sole assistant (as was the case at the commencement) was some devoted person, generally an old stupid creature, whose life was not considered by the runaways as worth preserving.

Often in one of these wretched hovels, where I could scarcely stand upright, have I been obliged to go my rounds to the straw beds of four or five individuals stretched in all the horrors of cholera on a damp floor, to give them both medicine and nourishment, and frequently delayed a long time for the mere want of a spoon in which to give them. Obliged, then, to go through the several operations of killing, skinning, and committing to the saucepan, a chicken to supply them with broth, which I have been obliged, for want of a messenger, to carry from house to house. I could easily enlarge upon these various vexations, but will only add that, when it is further considered that the practitioner has to go through such a routine as this several times in the day, at places remote from his residence, and from each other, and in so doing to travel over roads scarcely deserving the name, perhaps, in bad weather, it will not, I think, be denied that it requires no small stock of perseverance to struggle against such a tide of opposing circumstances.

Of the cases, 154 were males, 206 females; of the deaths, 71 males, 91 females. Of the cases which fell to my lot, I was unable to take notes of more than 103; many died before I could reach them, and to others I could not go at all.

These cases were as follow:—

| Age. | No. of Cases. | Recoveries. | Deaths. | Deaths in Collapse. | Recovered from extr. Collapse. |
|---|---|---|---|---|---|
| Over 50 | 23 | 7 | 16 | 9 | 0 |
| 40 | 8 | 3 | 5 | 5 | 0 |
| 30 | 18 | 13 | 5 | 0 | 4 |
| 20 | 20 | 14 | 6 | 6 | 4 |
| 10 | 13 | 8 | 5 | 5 | 4 |
| 5 | 14 | 3 | 11 | 9 | 2 |
| 1 | 14 | 8 | 6 | 4 | 2 |

Hence it will appear, that, in forming a prognosis, the age of the patient is an important element of the calculation; and is also one on which, in fatal cases, will greatly depend the period of the disease at which death ensues.

Thus, we see that above the age of 40, there was no case of recovery from collapse in the second degree, or extreme collapse; while, as we descend, we find that, within certain limits, the powers of resistance or re-action increase. Thus of 13 recoveries between the ages of 30 and 40, four had been cases of extreme collapse: from which we may conclude that the age at which there is, cæteris paribus, the best chance of recovery is that between 10 and 40; those above the one and below the other, being seldom able to withstand the consecutive fever ensuing on the state of extreme collapse. For the convenience of detailing the treatment, I will divide the disease into three stages; viz. the 1st, or premonitory; the 2nd, or that of collapse, in the first degree; and the 3rd, or collapse in the second degree.

*First, or Premonitory Stage.*—This, in a majority of cases, consists in what would, in ordinary times, be called simple diarrhœa, with the addition, sometimes, of an occasional cramp, and an absence or diminution of bilious colour in the discharges, sometimes there are merely borborygmi. If there be no diminution of bile in the fæces a commanding dose of tinct. opii, *e. g.* gtt. lx. (to an adult), will frequently arrest the disease; but if the secretion of bile be also diminished, we should premise cal. gr. iii., op. gr. ii., and in the course of an hour or two (if necessary) give the laudanum. If nausea be also present, the addition of a few drops of ol. menthæ pip. will be advisable. This form of premonitory stage, exists sometimes for several days without advancing further, being occasionally so slight as to attract little or no attention; and such cases have been by some authors set down as the most dangerous; but I have found it otherwise, and, indeed, would à priori expect to find it so; for the symptoms becoming, as it were, stationary on the threshold, imply a power of resistance to the inroads of the distemper. In some cases there is at first no affection of the bowels, but merely a general feeling of malaise, conveyed by the people here in the vague expressions of a "great weight about the heart," with an unaccountable degree of languor, depression, and weak pulse. This, in my experience, has been the most rapid and uncontrollable form of the disease, and generally occurs in persons worn down by previous apprehension about it; as by unrelieved attendance on the sick. Vomiting, purging, and cramps come on almost simultaneously, and carry off the patient in three or four hours. In this case, I have found the following *formulæ* very useful—℞. Camphoræ ℨi., tinct. opii ℥ss., ol. menthæ pip. gtt. xvi., tinct. cardam. comp. ℥iss. solve. A drachm of this tincture may be given in any agreeable vehicle, and has, I think, rescued many from impending cholera. More rarely the first symptom is vomiting, in which case, it being probable that there are some offending matters in the stomach, an emetic is the most advisable remedy. Most rarely of all does it happen that the disease begins with cramps; and here the combination of camphor, opium, and capsicum, sometimes acts like a charm.

*Case.* A man, aged 60, at the burial of his wife, who had died of the disease, drank two glasses of raw whiskey on an empty stomach. Shortly afterwards, he was attacked with most violent cramps of arms, legs, and thighs, hurried breathing, jactitation and nausea, but no vomiting nor purging. He had also the decided cholera expression of eye. I gave him 4 grains of opium, 10 grains of camphor, 3 grains of capsicum, and 4 drops of oil of peppermint, followed by a cordial draught, and ordered heat to be applied to the extremities and spine. In the course of a few hours copious perspiration, followed by sleep, came on; and he was well. In all cases it is most important that the patient should be at once placed in bed, as nothing is more injurious in every stage of the disease than the exertion of gaining or keeping the erect posture, and it has happened that they have died in the effort. The usual means for preserving or restoring a proper temperature, should at the same time be resorted to. If the treatment above recommended should not arrest the disease, and diarrhœa continues or supervenes, or where there is, in addition, an irritability of stomach, forbidding the use of remedies by the mouth, an enema, containing plumbi acetat. ℈ii., tinct. opii ℨi., ought to be immediately administered; and this invaluable remedy is the sheet-anchor also in treating the more advanced stages.

*Second Stage, or Collapse in the first degree.*—By this I would be understood to mean that state in which there are vomiting and purging; the heat, though diminished, not being wholly extinct, and the skin dry, or at least not covered by a cold perspiration: there is also the sunken eye and hollow voice, the cold tongue and breath, and absence of pulse, with or without intense thirst and cramps. The latter, though generally attendant, is not always. In some old debilitated subjects the vis vitæ seems so completely extinguished as to be incapable of the abnormal nervous action. Neither is the danger always in proportion to their severity when they do occur.

As to the prognosis in this case, we may state generally, that if matters remain stationary for six or eight hours, this will be favorable. Within that time, in such cases, warm perspiration commonly sets in, purging wholly in great measure ceases, water is passed and vomiting is superseded by nausea and eructations, in short, a mere irritable stomach amenable to ordinary remedies. And with respect to vomiting in general in this disease, I have long learned to look upon it as by no means *the* formidable symptom. On the contrary, I consider it as an indication of a certain vital energy remaining, and have seen cases terminate most favourably where it has continued incessant all through. I accordingly never think of endeavouring to check it directly; nay, where it has ceased before reaction has begun to set in, and where, the thirst continuing, a quantity of fluid is accumulated in the stomach and oppresses the respiration and the general system, I am sure it is advisable to re-establish it. The following is a case in point.

Daniel Hanly, æt. 60, an asthmatic of long standing, 13 hours ill. *Symp-*

*toms*—vomiting, purging, cramps, &c. Has taken plumbi acet., capsicum and opium, with fluid stimulants, which were all rejected. Vomiting has ceased for some hours, during which he has drunk a great deal, chiefly water, and has retained all. Complete collapse—no pulse—cold surface—respiration much impeded, and great fullness of stomach. I gave him a mustard emetic, which operated immediately, and caused the discharge of above a gallon of fluid. The improvement which instantly followed was very marked. The pulse became distinct, the respiration easier, and, though he had been apparently almost moribund, he rallied completely, and for some days seemed likely to do well, but subsequently sank in the consecutive fever.

*Treatment.* The great object to be kept in view in this, as in every other stage of the disease, is the checking of the diarrhœa, and, for effecting this, I know of no means so effectual as the injection of acet. of lead and tinct. opii, as above directed; observing that the quantity of water used should not be large, (about 4 oz.), to avoid mechanical stimulation of the gut. The strength must be supported by weak chicken-broth, thin arrow-root, sago, and wine in small quantities at short intervals. The best stimulants are, I think, preparations of ammonia. The spirituous stimulants must be given very sparingly, and only in a dilute state. It was here that the most capital errors were committed in the treatment of the disease on its first appearance in these countries. I was myself, among others, entirely led away by the idea that the most powerful stimulants ought to be employed for rousing the system out of a state of such extreme prostration, not reflecting that the blast which gives intensity to the furnace will extinguish the flickering taper. To shew the fatal effects of this wholesale stimulation I will quote a case.

A hale man, 60 years of age, was attacked on the night of the 9th November, 1837. He got some pills containing camphor, capsicum and opium, followed by a draught of hot, strong punch. About 12 o'clock at noon, the day following, when I saw him, the pulse and heat had returned, and he was inclined to sleep, but when roused there was a certain wildness of look and manner about him, for which I could not at the time account. His friends, as I afterwards discovered, had given him two tumblers of punch, which not then knowing, I did not warn them against such a mode of proceeding. I left him, fully confident that healthy reaction had been established, and that he was in a fair way of doing well. In an hour after he was dead; and on enquiry I learned that, after my departure, he asked for a glass of pure brandy, got it, sat up in the bed, drank it off, put up both his hands to his head, and died.

I candidly acknowledge that I formerly did a great deal of mischief by indiscreet zeal in this respect. Whatever difference of opinion there may be about other confessionals, a medical one would, I conceive, be of infinite service to the profession and society at large; and if some man of extensive experience and high character (for even such have made mistakes) would publish his "errors," such a medical chart would warn off from dangerous rocks and quicksands, and prevent many a wreck. Of "successful cases" there is no lack—we are pretty sure to hear of them, while those of an opposite description are commonly included in an act of general amnesty. The spirit. ammon. aromat. is one of the mildest and best diffusible stimulants we can employ, being free from the effects on the sensorium, which form the great objection to the spirituous; indeed the use of ammonia contemporaneously with spirituous stimulants exercises a control over this deleterious property—℥j. of the spt. ammon. arom. may be diffused through ℥xij.—xiv. of water, and half a wine-glass given every quarter of an hour; its increase, diminution, or suspension being of course regulated by the effects produced. It is of the last importance that the most unremitting attention be paid to the support of the strength by light nourishing drinks, as before observed; and also that the patient should be prevented from assuming the erect posture, than which nothing more exhausts the feeble powers that remain. I have, in addition to these, always given calom. gr. j., cret. ppt. gr. iij., capsici, ½ gr. every second hour, with a view to restoring the secretions. Here also the external means of restoring heat should not be neglected, but excess is as carefully to be avoided as in the matter of internal stimulants, and I am sure I have seen mischievous results from overacting this part also. The application of tin vessels containing hot water to the soles of the feet and calves of the legs seems sufficient. Sinapisms to the chest and epigastrium are also advisable, particularly in those cases where there is a great degree of præcordial oppression; and they should also be applied to the back of the neck and calves, where, on the establishment of reaction, there appears a tendency towards affection of the head; and this should most promptly be attended to, in the case of children especially.

*Consecutive Fever.*—As before observed, this stage generally terminates in a state which scarcely deserves a more formidable epithet than simple fever, or, at farthest, the mildest form of typhus; there being merely a tongue coated white, quickness of pulse, flushed face, and no further evidence of a local determination than the irritability of stomach before alluded to. which, though often obstinate and troublesome, is not dangerous, there being no evidence of gastritis; and the symptom generally yields to magnesia and rhubarb in peppermint water and effervescing draughts, and seldom requires even the application of a blister. Sometimes however it proves more lingering, seems to have no tendency to termination by crisis, commonly so called, and must be treated on general principles. It must be understood that the above doses are for adults, and this must be borne in mind all through these remarks.

*Thirdly, Collapse in the Second Degree.*—In this are superadded to the last group of symptoms complete loss of heat, the surface being covered with cold clammy perspiration. The voice is sunk to a whisper. In some cases there is a cessation of the cramps, in others they continue and extend to the trunk, frequently the region of the stomach, (a very fatal symptom). Sometimes an intolerable præcordial oppression amounting to agony, from which the wretched victim calls on death to release him. Sometimes there is a grumous diarrhœa, which also is a very fatal accompaniment. The thirst in some cases is intense, in others not at all so—and very frequently the drinks are all retained, the stomach having as it were lost its sensibility. The patient lies in a lethargic. state, his eyelids partially closed, and exhibiting the white of the eye, the conjunctival vessels of which are sometimes slightly injected—when roused from this state his intellect is quite unimpaired, and remains so till within a very short time of the closing scene. This state of things may last from three or four hours to as many days, but the latter is rare. This is a condition from which no aged person and but few children will be found to recover, because the consecutive fever which ensues upon this aggravated form of collapse is uniformly severe, and neither of these two classes of patients has stamina to withstand it.

*The Treatment* of this stage is substantially the same in principle as that of the foregoing—the urgency of the danger enforcing a stricter attention to the several particulars above detailed, and particularly as regards stimulation. In the consecutive fever after this stage the typhoid type is very marked indeed. In old people and adults there is seldom any particular local affection discernible, but in young children the head is almost universally engaged, and the symptoms come on so rapidly that nothing but the most prompt and vigorous measures will be successful; even an hour's delay in the recognition and treatment of them may prove fatal. "Venienti occurrite morbo" is in no case a sounder maxim than in this.

*Case.—March 28th*, 1838, (10½ a. m.) Joan Owney, æt. 4—ill ten hours. Is just rallying from extreme collapse. A sinapism has been applied to her chest, and an injection of starch and tinct. opii has been given. Vomiting and purging continue, but less—pulse and heat returning; lies with eyes exposed and turned up in the sockets. Has taken 1 gr. calom., cret. ppt. gr. iij., op. gr. $\frac{1}{10}$, also a little weak cordial, and a little mist. camphoræ, with a small quantity of laudanum. 2 *o'clock, p. m.* Coma rather less—stomach quiet, but purging of white matter continues. I ordered stimulants to be stopped, put a blister to poll, and had the starch injection repeated with the addition of plumbi acet. gr. vj., tinct. opii, gtt. xxx., the whole amounting to f. ℥ij.

*29th, Noon.* Blister has risen well. She is still drowsy, but sensible when roused—eyes clear, pupils contracted—heat not above natural—bowels confined —no urine. Sumat statim calomel, gr. v. Necnon calom., p. jacobi, ā gr. j. 2dis horis. Sinapisms to calves of legs—stop chicken broth—drink whey.

7½ *o'clock, p. m.* Is sleeping naturally—sinapisms acted well—pulse 112. Passes flatus from bowels, which are confined and sound, tympanitic—injiciatur enema fætidum—pergat.

She continued to improve, and eventually recovered, having passed by mouth on the 31st a long worm alive—a circumstance very frequent in these patients.

I will relate another case to shew the insidiousness with which this fatal form of consecutive fever sometimes makes its attack when matters appear to be going on favourably.

Michael Shea, æt. 4, was attacked early on the morning of 12th April, 1838. I saw him at ½ p. m. in a state of extreme collapse. I ordered an injection of plumbi acet. gr. v., tinct. opii, gtt. xxx. Applied a sinapism to epigastrium and nucha, and gave calom. gr. ½, cret. gr. j., capsici, gr. ½.

2 *o'clock, p. m.* Reaction commencing—pulse sensible—head wet with warm perspiration—heat generally returning—enema was retained. Wishes for cold water. Has taken two of the powders of calom. capsic. and cret. Eyes turned in sockets, but clear and bright when roused. Voice strong. 6 *o'clock, p. m.* Same state, except that vomiting and purging have ceased. Applicat. vesicator nuchæ—sumat. 3tis horis calom. gr. ¼, cret. gr. j.

*13th*, 3 *o'clock, p. m.* Blister acted well. Appears to sleep naturally—is peevish when disturbed—countenance natural, except a slight remnant of cholera expression of eye—skin cool—tongue moist, yellowish. A slight discharge of greenish fluid from bowels, which are tender and tympanitic. Took this morning calom. gr. iij., p. antim. gr. ij.—injec. vesperi enema fætidum. 14th. Seems quite easy—sleeps a good deal, but is restless in it—skin natural. The injection brought away nothing but some greenish watery fluid. Two worms were discharged to-day, one by mouth the other by stool, the former alive. Belly continues tympanitic. Tongue moist. Passed urine to-day.

From this until the 16th he seemed to improve, when he took a decided turn for the worse. He became quite comatose. It was impossible to rouse him. His pupils at first used to contract to light, but were afterwards permanently contracted. He used to grind his teeth, and his bowels were obstinately costive, except that an enema occasionally brought away some slimy fæces. Blisters and sinapisms were tried to no purpose, and he died on the 19th, at 10, p. m.

I will now, in conclusion, take a cursory review of the remedial agents which have been used by myself and others in this disease. *Bleeding.*—I have not seen in the course of the late epidemic any case of the actual disease in which it was at all indicated but one, viz. the first of the cases which I have recorded. It was not practised in that case, and the patient recovered. The only stage of the disease, I think, in which any reasoning physician ought to think of it, is the premonitory, and only in that rarest form of it in which there are violent cramps, no diminution of heat, with a certain degree of excitement and strong pulse. In the consecutive fever it is otherwise, but even there must be adopted with caution. I saw only one case in the late epidemic where I thought myself justifiable in recommending it; it was a case where there was a decidedly active determination to the head, with delirium—the subject a strong man. It was not submitted to, and he also recovered.

*Opium* may be given by mouth in the premonitory stage freely, in the first stage of collapse more sparingly by the mouth, but freely in enema, as elsewhere recommended. In collapse in the 2d degree, if the discharges have ceased, it is madness to give it in either way; if not, it may be given in enema, not only without danger, but with the greatest advantage. There is however a combination of it with camphor, the formula of which I have before given, which I have used in every stage without (as far as I have observed) any injurious effects in any. In those cases which, though properly designable at first by the term "cholera-phobia," yet imply a state of nervous predisposition to the disease that often ultimately glides into it, I have found it most useful; and even in the extreme collapse, where the discharges have ceased, it sometimes appears to restore to the stomach its excitability, the administration of it being in many instances quickly followed by a copious discharge of fluids, which for a considerable time previous had remained a useless incumbrance to the organ. It is in my opinion a very useful medicine to keep at hand, during the prevalence of an epidemic, in families and in districts where, from their extent and remoteness, the physician must of necessity often prescribe without seeing the patient.

*Acetate of Lead.*—I cannot speak very highly of this medicine given by mouth. I have tried it very extensively in combination with capsicum and opium, and do not think that it exercises any control over the decidedly malignant form of the disease. It is a most powerful medicine in sporadic diarrhœa which has resisted other remedies, and, as there are many cases of this mixed up with those of cholera in the course of an epidemic, I suspect that, from its acknowledged efficacy in them, it mainly (I do not take it on me to say entirely) derives the high character which some eminent physicians have conferred upon it. In the form of enema it is however invaluable.

*Calomel.*—This in small doses is advisable, not as exercising any specific power of rallying from collape, but as tending to restore the secretions. It was given in truly *heroic* doses in the early treatment of cholera in these countries, and it will be remembered that the current aphorism was "salivate your patient and he is safe." But I may here remark that in no case of recovery from it, though I universally employed calomel, was there the least appearance of ptyalism.

*Cold Water.*—This has been extolled at different times as a remedy of great value. The true state of the case, however, I conceive to be simply this. The cases in which it is so anxiously desired and so copiously drunk are in general those in which a considerable degree of vital energy remains, evidenced by the intense thirst and constant vomiting, and which would recover as well under the use of other judicious means. One thing is certain, that it does no harm, and it is pleasant to be able to gratify the sufferers in this respect without prejudicing their chance of recovery.

*Emetics.*—The utility of these I am disposed to limit to that form of premonitory stage in which vomiting is the first symptom, and that form of collapse in the second degree which I mentioned before, in which there is an accumulation of fluid in the stomach. *Stimulants.*—I have already stated my views respecting these. *Sinapisms.*—These are useful in a twofold point; 1stly, for helping to rouse the dormant sensibility: 2dly, for counter-irritation, where local determination impends. They act more speedily than blisters, and are therefore so far preferable.

*Nourishment.*—This is a point of primary importance, and I believe that much of the mortality which prevailed in the early treatment of the disease in this country was owing to practitioners trusting too much to medicine, and vainly seeking after some specific, while they suffered this to be neglected.

The patient ought to be abundantly supplied with diluent drinks, and small quantities at a time of light nourishment of the liquid kind. The success of the case depends as much, I would say, on the diligent administration of these, in conjunction with other matters, exclusively the province of the nurse-tender, as on the skill of the physician; indeed, without the cordial and efficient co-operation of the former, the best efforts of the latter must prove nugatory and end in disappointment.

Having extended these remarks to a much greater length than I had originally intended, I will now conclude, expressing a hope that the hints here thrown out, may not prove altogether useless to those who may be hereafter called on to treat this most formidable malady.

I remain your obedient humble servant,

EDMOND SHARKEY, M.B. T.C.D.

---

### HASLAR HOSPITAL.

### A CASE OF PERFECT ANCHYLOSIS OF THE FIVE SUPERIOR CERVICAL VERTEBRÆ TO EACH OTHER, AND COMPLETE DISLOCATION BACKWARDS, OF THE FIFTH FROM THE SIXTH, WITHOUT FRACTURE. By *Stephen S. Stanley*, Assistant Surgeon of Haslar Hospital.

*History of the Case sent with the Patient; with the subsequent Symptoms, and Treatment pursued by Dr. Mortimer, the Senior Surgeon, under whose care he was placed.*

George Weldon, æt 37, seaman, lost his footing yesterday evening, the 20th of July, 1838, about nine o'clock, and fell backwards, on his head, on the deck. Found him immediately afterwards complaining of a severe pain in the back part of his neck, and between the shoulders, and of pain and numbness in the arms. His face was pale, and his pulse weak. Five grs. of Carb. Ammonia in an ounce of camphor mixture was administered—after which he rallied. He is worse this morning, complaining now of numbness, not only in the arms but also in the legs; of the pain in the back part of the neck being more severe, and of inability to turn or move in any direction. As the ship is in dock, it is thought advisable to send him for the benefit of hospital treatment.

(Signed) JAMES M. DEAS,

July 21st, 1838. Assistant-Surgeon, H.M.S. Pique.

Admitted into the Haslar Royal Naval Hospital, on the 21st of July, 1838, at eleven A.M. in a state of perfect consciousness, no wound, no external appearance of bruise. Both arms are commencingly paralytic, the left the most so—the accident occurred yesterday evening, since which he has not passed urine, nor have the bowels been opened; the pulse is slow, weak and oppressed. Pupils unaffected, nor does he refer to any complaint, save about the muscles of the neck and shoulders.

His breathing is undisturbed, catheter introduced, haustus sennæ statim, enema. Evening: a free evacuation of the bowels, pulse up and sharp, enema repr. v. s. ad. xx ounces. 22d July: Respiration is hurried, the pulse is weak, there are continual attempts to expectorate a frothy mucus, but the attempt is ineffectual—is most anxious to inhale air, he desires to be raised higher—higher still—this is accomplished with facility, by the rude yet excellent apparatus of Borthwick, repr. enema—Catheterismus, hyd. submur. grs. vj., pulv. jalap. a scruple, syr. q. s. ft. bolus statim. Noon:—He is easier, he breathes more uninterruptedly, but the pulse flags. Evening:—Respiration laborious, death is approaching. Died at half-past four o'clock on the morning of the 23d, exactly fifty-five hours and a half after the accident, and forty-one hours and a half after his admision into this Hospital.

Post Mortem.—On the posterior surface of the body, extending from the occiput to as far as the 6th or 7th dorsal vertebra, there was considerable ecchymosis; and in making a section of the integuments and subcutaneous cellular tissue, a quantity of blood was found effused into its texture. In prosecuting the dissection further, especially in a space reaching from the 1st cervical to the 2nd dorsal vertebra, coagulated blood in great quantity was found surrounding the muscular fibres, a number of which were ruptured and softened; these being sponged away, a little more careful dissection exposed to view a considerable displacement *backwards*, of the 5th from the 6th cervical vertebra. All the blood was sponged and cleared away, and as much of the soft parts removed as was possible, for the purpose of ascertaining the exact position of the dislocated vertebra. It was then found that the little finger could easily be passed, underneath it, into the spinal canal; that the body of the 5th pressed severely on the spinal cord, and rested on the lamina and spinous process of the 6th cervical vertebra. The spinal column was now removed (sawing through the angle of the ribs) at the 7th dorsal vertebra. It was then ascertained, beyond all doubt, that the injury was a complete dislocation, *without fracture*. The ligaments and intervertebral substance were all ruptured; and, when suspended from above, the parts were held together by the vertebral arteries and spinal marrow, with its theca alone; the theca vertebralis being uninjured.

Head.—The cranium thick, and very heavy; the thinnest part measuring 2¼ lines, and the thickest 5½ lines. The great longitudinal sinus was gorged with blood, and so large as to admit with ease the forefinger. The medullary substance of the brain was soft and very vascular: when the section of the centrum magnum ovale was made, it was found studded with spots of red blood; nothing else was observed until an attempt was made to remove it; it was then found impossible to pass a knife through the foramen magnum, to make a section of the medulla oblongata. The brain was, however, removed; and it was then ascertained that the foramen magnum was so much contracted, as scarcely to admit the point of the little finger. On closer inspection, and after dissecting off the dura mater in this situation, the constriction was evidently produced by the odontoid process of the axis being much larger than natural, and projecting, in a conspicuous manner, upwards towards the base of the brain, and backwards on the medulla oblongata, which, from the little that was attached to the pons varolii appeared small, and nearly flat. By applying the saw at the posterior margin of the foramen magnum, and carrying it obliquely forwards and upwards, a section of the base of the cranium was now made for the purpose of ascertaining the exact condition of the odontoid process, and the beautifully arranged ligaments in this situation. The section being completed, and a little dissection made, it was ascertained that the whole of the cervical vertebræ, from the atlas down to the seat of dislocation, were completely anchylosed. Not the least vestige of ligamentous structure could be observed, with the exception of the capsular, and occipito-atlantal ligaments, forming the articulation between the occiput and atlas; and of these, the capsular ligaments and synovial membranes, when cut into, were found to be so much thickened and altered in structure, as more to resemble cartilage than ligament, and calculated to impede seriously, if not altogether, the nodding actions of the head, and slight lateral motion, which this articulation permits. No trace could be found whatever of the apparatus ligamentosus, and lateral ligaments, connecting the occiput with the atlas; neither was there anything remaining in the form of the ligaments, which complete the articulation between the atlas and axis: but nature, ever bountiful, had formed a beautiful provision for the absence of the transverse ligament, by an isthmus of bone, extending from the anterior aspect of the odontoid process to the posterior concave surface of the anterior arch of the atlas:—thus, in most respects, answering every purpose for which the transverse ligament is known, although placed in a situation diametrically opposite.

After the usual process of maceration, the bones appeared white, and exceedingly compact in their tissue, and, with the exception of their anchylosed condition, perfectly normal—their form in every respect not appearing to differ from the general characters by which these vertebræ are known.

The most remarkable feature in the whole preparation—and evidently the result of a former dislocation forwards—is the position of the atlas; which, on the right side especially, is pushed forwards and upwards from the articulating surface of the axis, so as to cause the odontoid process to present itself nearly in the centre of the circle of the atlas. A bridge of bone, exactly half an inch in length, and varying from three to four lines in breadth, passes nearly horizontally forwards, from the odontoid process to the atlas, as described above, and connects them together. The axis is also pushed forwards in the same manner from the third cervical vertebra, but not to so great an extent, giving the entire preparation a twisted appearance to the left side. Its length measuring anteriorly, from the superior margin of the ring of the atlas to the inferior margin of the body of the fifth cervical vertebra, is 3½ inches. The diameter of the spinal foramen of the atlas, from behind forwards, is exactly one inch and four lines, and the transverse diameter one inch and half a line. The odontoid process, instead of terminating at its apex in a point as it generally does, presents a broad and irregular ovoid form, measuring, transversely, half an inch—and from behind forwards, including the bony ridge alluded to, one inch; its length is three-fourths of an inch, and its distance from the posterior arch of the ring of the atlas only four lines.

REMARKS.—It may be supposed that, having ascertained the exact nature of this accident, the author of this paper was very anxious to obtain every particular relating to this man's history; and as the *Pique* was still riding at Spithead, he took the earliest opportunity of going on board, for the express purpose of gaining all the information possible; and he has to thank Mr. Deas, the assistant-surgeon, for his kindness in furthering his views on that occasion.

It appears that the man had, for some years past, always been subject to a stiff neck, that he very often complained of rheumatic pains in that region, and of sore throat. He was, nevertheless, a very efficient and active seaman, always doing his duty, and never on the sick list; but was unable to move his head to one side, and was compelled to turn his whole body round when he was desirous of looking either to the right or to the left. It further appears that, at the time the accident occurred, the deceased, although not drunk, was in the state that sailors call "rather fresh," and was "larking" with some of his messmates, and in attempting to catch one of them, his foot slipped, and he fell backwards, his head only SLIGHTLY striking the deck.

*Note.*—Mr. Stanley has entered into some research for similar cases, and has appended short notices of them to his paper; but we have not space for them here. We hope the medical officers of our naval and military hospitals will not allow the valuable facts that occur in these noble institutions, to slumber in comparative oblivion among the records of the past.—(*Ed.*)

### ADDENBROOKE'S HOSPITAL.

### Clinical Contributions from Addenbrooke's Hospital, Cambridge, for the Years 1836-37. By H. J. H. Bond, M.D.—(Continued.)

### Diseases of the Stomach and Intestines.

The cases of diseases of the stomach and intestines, taken collectively, amounted in the years 1836-37, to 13.89 per cent. of the total admissions during that period: the respective aggregates of cases entered as affections of the stomach and as affections of the intestines being nearly equal. The data for determining the proportion of male and female, and of town and country cases exist only for the year 1837, from these it appears that 9.03 per cent of the male, and 20.12 of the female admissions in that year, and 13.68 per cent. of the cases from the country and 16.25 per cent. of those from towns appertained to this class of affections.

### Diseases of the Stomach.

Cases entered in the registers under this title during the two years, amounted to 6.69 per cent. of the total admissions; and in the year 1837, 3.67 per cent. of the male, and 9.54 per cent. of the female cases were of this description.

The results of an analysis of the cases of stomach affections under the charge of the author, and of which the notes were preserved, seemed to correspond best with that classification of gastric disorders, in which they are referred to the two following divisions—the 1st, in which the gastric disturbance is unaccompanied by any excess of blood or augmented circulation in the stomach, and the 2nd, in which lesion of the gastric circulation, from simple congestion or hyperæmia to inflammation of the mucous membrane, including the products of such lesion, viz. disorganization of the gastric tissues, constitute the principal element of disease.

The reference, however, of each case, to one or other of these divisions was not so much determined by the absence or presence of particular symptoms conceived to be expressive of vascular derangement of the stomach, as (in accordance with the less exclusive views which have prevailed since the doctrines of Broussais have received the correction of more general experience) by the predominant and collective character of the symptoms; and more especially by the result of the modes of treatment employed, the real touch-stone, as Andral expresses it, of the nature of the disease in this class of affections.

*First Division of Gastric Affections, or Cases of Functional Derangement of the Stomach, unaccompanied by any Symptom denoting Excess of Blood, or Excitement of the Circulation in that Organ.*

*General Statement.*—The cases in the general practice of the hospital, registered under the title of dyspepsia, making allowance for some latitude of diagnosis, were probably for the most part of this description, and the following is the numerical statement of them—5.96 per cent. of the admissions in the two years were entered as cases of dyspepsia: 3.08 per cent. of the males, and 8.42 per cent. of the female patients being dyspeptics, also, in the year 1837, the proportion of town and country cases of dyspepsia to the respective totals of town and country admissions being nearly equal.

*Analysis of Cases.*—In the present division are included 101 cases under the author's care, in which the principal morbid phenomena referrible to the stomach consisted in impairment or perversion of its digestive functions, with derangement of its sensibility—admitting of a further sub-division, according as in addition to these states, there existed perversion of its muscular actions, producing frequent retching or vomiting, but still unattended by any other symptom denoting vascular excitement. It is probable that a still further distinction of cases in reality existed, in reference to the absence or presence of morbid secretions of the gastro-mucous membrane—but there was no certain guide in the symptoms to denote this distinction; or at least, such a state of the secretions, if recognizable, appeared incidental to any form of the disorder; whereas the occurrence of vomiting as a frequent and prominent symptom seemed to mark an affection of a nature distinct from the other more simple form.

*Impairment or Perversion of the Digestive Functions* was in these cases denoted by,—1st. Derangement of the appetite, which was present in more than two-thirds of the 101 cases, either as total anorexia with aversion to food, or diminution of appetite with disrelish of or indifference for food, or capriciousness of the appetite shown either in the choice of particular articles of diet, or in the alternation between a desire and disinclination for food, or lastly, a more or less constant craving for food, its ingestion not being followed by satiety.

2dly. Excessive development of gas in the stomach; which occurred in nearly a third of the cases, either indifferently during fasting or digestion, or at various intervals from immediately after ingestion of the food, till the completion of the gastric digestion—this symptom occasionally was the most prominent and urgent of any.

3dly. Disturbance of the functions or sensibilities of other organs supervening or becoming aggravated during digestion.

*Derangement of the Sensibility of the Stomach.*—Granting that pain or disordered sensations referred to various regions, but all in the vicinity, in the stomach and for the most part connected with other morbid phenomena plainly originating in the stomach, denote modifications of the sensibility of that organ; this was by far the most constant morbid condition met with, as it is recorded in 87 out of the 101 cases: with regard to its locality, the most frequent was the epigastrium, next in frequency the left hypochondrium, and nearly as frequently the sternum, especially towards its lower extremity; in connexion with pain in one or other of those situations there occasionally existed uneasy sensations or positive pain in some parts not corresponding to the stomach and beyond the abdomen, as between the scapulæ or the portion of the back opposite the epigastrium: in one instance the most urgent complaint was of pain referred constantly to the *vertebra prominens*, which disappears with the gastric disorder.

With regard to the morbid character of the sensations felt in these situations, the epigastrium more especially, while a considerable number complained simply of pain, others again assigned some peculiar character to the morbid sensation, such as aching, smarting, gnawing, nipping; in some the attacks were violent and spasmodic; in others there was a sensation of weight; in others again of heat; in a few on the contrary of cold in the region of the stomach; others again denied having any pain, and complained only of an indefinable uneasiness or distress at the epigastrium. But these modifications of sensibility seemed to occur indifferently in any of the varieties of the disorder, and perhaps were rather different modes of describing pain, than real differences of sensation. One only of the characters assigned to the lesion of sensibility deserves more particular mention, both from its very frequent occurrence and from the direct evidence of its being referrible to the stomach, and this was the sensation of *sinking*—this, it is well known, is the most common expression given by a large proportion of invalids to the sensation they experience at the epigastrium; and Dr. Beaumont states that this was uniformly the sensation felt by Alexis St. Martin, when the tube was introduced by the external aperture into the stomach during its empty state, and when the gastric juice flowed in more than ordinary abundance. Besides these various morbid sensations, which frequently occurred independently of ingesta in the stomach, and sometimes indeed, were relieved by ingesta of any kind, there was the additional symptom in many of a sense of load or oppression after a meal of ordinary or less than ordinary quantity. One instance may here be alluded to, of a singular description of morbid sensibility, evinced by an uncontrollable avidity for drinking cold water.

Sensibility to pressure in the epigastrium, seldom amounting to tenderness but still more than is natural, was present in about a sixth, excluding those cases in which this might have been produced by the efforts of vomiting: leeching and counter-irritation was not found to be more frequently of service in these instances than where such symptom was absent, which would intimate its independence of the vascular condition of the stomach. In several cases the patients complained of a fluttering or pulsation at the epigastrium or left hypochondrium, and occasionally such pulsation was perceptible to the touch.

*Conditions of other Portions of the Digestive Canal.*—Next to the symptoms proceeding from the conditions of the stomach itself, may be noticed those appertaining to other portions of the primæ viæ.

And first with regard to the tongue: for the most part its appearance was rather typical of the general state of the patient's health, and to be viewed as a complexional feature than taken as an index of the immediate condition of the stomach: since it was rather in its form, textural colour, and degree of firmness, its tension, flabbiness, or tremulousness than in the varieties of its secretions, that it was included within the range of morbid phenomena; and its return to a normal condition was not so often coincident with the removal of the actual gastric disorder as contemporaneous with the more gradual restoration of the other elements of health, as of the complexion, embonpoint and muscular strength: indeed, in many instances, it seemed to be the most reluctant to yield of all the morbid states, and to persist when the return to health in every other respect was complete. It may be added, however, that whereas, in this division of cases (from which those are excluded in which increased vascularity or inflammatory action of the stomach was supposed to have been present) those appearances of the tongue denoting, as it is believed, an inflammatory state of the stomach, as red and elevated papillæ, general redness, or a central streak of redness, were very seldom though occasionally observed. On the contrary, paleness of the surface was very frequently noted, and instead of dryness, more often an excess of moisture or halituousness: and whereas a thick dirty white fur is the most ordinary condition of the tongue when hyperæmia or inflammatory action of the stomach occurs; under the present form of disorder a slight, slimy, mucous covering, or merely a whitish appearance of the villi without any secretion of fur, was for the most part remarked. The condition of the gums and the mucous membrane of the mouth and fauces generally, like that of the tongue, was rather a feature in the general physiognomy of the disorder, than a symptom varying with the phases of the gastric derangement. The morbid sensibility of the stomach seemed occasionally to extend to the œsophagus, as from its nervous association might be anticipated: while in the peculiarity of its morbid sensations the nature of its function was still represented, as when the patient complained of a sense of choaking when nothing was present in the œsophagus; or of a difficulty in the passage of ingesta when there was no obstruction.

But proceeding in the opposite direction from the stomach, as the centre from which the disorder emanated, the remainder of the canal was in a more positive manner implicated in the functional disturbance—a necessary consequence, since any failure in the integrity of the gastric functions unavoidably entails upon the intestines an imperfection in theirs—but obvious as this is, the want of duly appreciating so simple an inference and rightly interpreting the nature of this aberration of the intestinal functions, has, it is to be feared, led to a serious mistake in practice, and an injudicious routine treatment been thereby adopted in the management of these every-day cases—in which, notwithstanding there is much room for caution and discrimination, if a speedy or secure recovery is to be effected, or an economical use of the dispensary be desirable. Allusion is made to the engrossing use of purgatives in every case in which torpidity of the intestinal canal is present; such a condition once or in any degree ascertained to exist, being immediately stamped as the " head and front of the offence," and made to suggest one invariable indication for treatment. But to return to the cases under consideration.

In the first place, other portions of the abdomen besides those corresponding to the region of the stomach, were not unfrequently the situation of pain or some morbid sensation without any accompanying inflammatory character, but which seemed to indicate either an aberration in the reference of gastric sensations to distant parts, or an extension of morbid sensibility to other tracts of the intestinal canal, or to the associated organs, as the liver or spleen : thus pain or a stitch in the right hypochondrium, spasms or aching in the umbilical, lumbar or iliac regions, were among the most ordinary complaints made by the patients, without their occurrence often furnishing any precise clue to the detection of functional disturbance of the subjacent organs. Sensibility to pressure was likewise, as in the case of the epigastrium, occasionally present in the hypochondria or in other portions of the anterior aspect of the abdomen, but not obviously connected with any alteration in the vascular condition of the viscera beneath. Pulsations also, either as internal sensations or recognizable by the touch, were met with, but rarely, in the umbilical region.

But if these symptoms of morbid sensibility existed independently of any inflammatory character or vasculaar congestion, it was seldom but they were accompanied by some derangement of the intestinal functions; though, as they did not generally subside with their returning integrity, they could scarcely be regarded as the result of such derangement. Unquestionably the most frequent form of intestinal derangement was costiveness, as this occurred in a noticeable degree in 50 out of the 101 cases, and either from its amount or duration was a prominent symptom in about a 4th. But that this condition of the bowels was but secondary to the gastric disorder, or its consequence and not its cause, was evident by its removal being though necessary to, yet not conclusive of the restoration of the functions of the stomach; indeed sometimes the measures found requisite to overcome the constipated habit, occasionally left the stomach in a state of still greater distress, to alleviate which an opposite mode of treatment and a different regimen were demanded; the sinking at the epigastrium being very often the residual ailment which by its irksomeness still maintained the patient in the condition of an invalid. In some, but comparatively few, cases, diarrhœa was the form of the intestinal disorder, while the gastric affection appeared the same as when constipation was its accompaniment. In about the same proportion of cases, alternations between constipation and diarrhœa, and in general an irritable state of the canal was associated with the stomach affection : but this was of more frequent occurrence where vomiting was the additional element in the latter disorder. Occasionally a spontaneous and smart diarrhœa relieved the stomach and led to recovery. Inordinate evolution of gas in the intestines was rare, which distinguished these cases from that form of hysteria in which this symptom is so common and is attended with impairment of the gastric function.

*Conditions of other Organs and Systems.*—This concludes the general statement and history of symptoms immediately connected with the stomach and organs functionally associated with it; but the ramifications of disorder were confined within no such narrow limits as these, but in many instances were coextensive with the entire organization, implicating more or less every system and function. It is from losing sight of this latter circumstance, viz. that the derangement of other and distant functions is but a consequence or extension of the original gastric disorder, (which is still retained as the substratum and course of the general disturbance,) that cases such as many of those forming the subject of the present analysis, in their course and progress, come to have a different and various nomenclature assigned them: whereas, if the present view be correct, they continue properly the same, the essence of the disorder being still the impairment of the gastric functions, and the adventitious derangement of the general health being merely its various phases—induced either by the nature of those external conditions, which as predisposing causes originated the gastric disorder, and at once impressed upon it that deleterious quality by which the whole organization speedily resents the mischief at the source of all healthy assimilation and nutrition—or resulting from the protraction simply of the gastric derangement and ensuing extension of its consequences. But this is to anticipate what will hereafter occur to be considered at more length: and mention is here made of this part of our subject merely as furnishing the apology for presenting the reader with a formal clinical communication on a topic so hackneyed as functional disorders of the stomach; which has been done on the grounds that many cases are practically as well as nosologically divorced from this class of affections, and have various names assigned them, as cachexia, anæmia, amenorrhœa, cephalalgia, and a separate department of therapeutics devoted to them, when essentially they are one and the same disease under different aspects and at different epochs.

*Permanent Disorders of other Organs and Systems resulting from and complicating the Gastric Affection.*

That the fact is as stated in the last paragraph, appears from the circumstance that in all the cases included in this analysis—in a large proportion of which the most serious amount of general disorder, confirmed cachexia with alteration of the tissues of the body, existed so long as to incapacitate the patients from following their occupations in life—the same gastric disorder, identical in its features with its simple form, was present from the beginning, continued throughout as an undercurrent but sustaining malady, and that its removal was the condition and first step in the general amelioration of the health.

To trace, independently of the merely sympathetic affections, what were the ulterior and permanent changes induced in other organs and systems, or, as in other instances, what was, at an early period even, the pernicious influence exerted by a malady, ordinarily so little alarming although so tedious, over the rest of the organization, will belong more properly to the exposition of the various predisposing causes and external conditions of the organization under which the disorder arose, and which rendered it productive of such results; but it may be proper here so far to touch upon this part of the subject as to state, that among these ulterior changes, the foremost appeared to be a disturbance of the circulation, originating probably in the impairment of the assimilating powers of the stomach, which at the centre of the vascular system produced a succession of morbid phenomena, such as distressing palpitation, aggravated dyspnœa, anormal sounds of the heart, oppression at the chest, faintishness or even syncope—in the course of the circulation, throbbing of the arteries—in its capillary termination, paleness or discoloration of the tegumentary or mucous tissues—and within the province of elimination, caused the serous habitus to be exchanged for more or less of serous infiltration or effusion in the cellular tissue of the face or extremities—and as a corollary of such disturbance of the circulatory functions, a want of firmness and tension in the solids of the body, or a loss of volume, if not emaciation. With this, a train of nervous symptoms, such as a more entire prostration of strength, with tremors of the tongue and lips, and hands; almost invariably distressing headache and vertigo; and a listlessness, more incapacitating even than weakness or positive suffering. As uniformly again impairment, in one form or another, of the menstrual function.

Such is a mere outline of the general disorder, induced by the derangement in the first instance of the gastric functions, in consequence either of its protracted duration or bad management, or from its having originated from such predisposing causes or under such pernicious circumstances in regard to habits, regimen, critical periods of life, and social conditions, as to impart to a malady, simple and controllable when not fostered by untoward accessories, an inveteracy and diffusiveness, capable of speedily producing baneful changes in the entire organization. The further illustration of these effects under especial agencies, resulting in the production of such conditions of the health, as have been distinguished conventionally by particular nosological terms, will presently be considered. In the mean time, as was stated in the early part of this communication, the occurrence of vomiting as a prominent symptom, seemed to denote a distinct form of the disorder, inasmuch, as besides the characteristics of morbid sensibility and impairment of the digestive functions, it imparted to it the attribute of extreme irritability, and the intolerance of the mere presence in the stomach of ingesta of any kind: but this as well as the other forms occurred equally when the constitution had not, as when it had become implicated, and while it indicated an increased intensity of the gastric affection, its occurrence did not seem materially to affect the chances or progress of further mischief in the rest of the œconomy; sometimes indeed it was surprizing how little disturbance resulted from its long continuance; in two instances a smart attack of bilious vomiting terminated the disorder: in two other instances however already alluded to, the severity of the vomiting and its association with intense headache were the grounds of apprehending some grave cerebral disease, which the issue alone disproved. Although it is to anticipate the subject of treatment, it may be here stated, that in one case the vomiting, when attended with headache, promptly ceased on cupping the occiput.

To return to the consideration of the implication of other and distant organs and systems in the disturbance of the chylopoietic viscera—a distinction may be here made *in limine* between the merely transient and sympathetic affections, and the real extension of functional derangement and lesion of nutrition to distant parts of the organization. The first it may be sufficient just to enumerate as the usual accidents in dyspeptic cases.

*Sympathetic Affections.*—The pulse, in those instances in which the affection had not made such inroads on the constitution as to have induced permanent derangement in the balance of the circulation with its ulterior effects on the organs and textures of the body, was still, more often than not, above its ordinary standard of frequency; in some instances the disorder had begun by an attack of ephemeral fever, and more often in its course was attended with a slight febrile movement at some period of the day; palpitation likewise was not a rare occurrence among the same division of cases.

Dyspnœa and cough generally unaccompanied with expectoration, were among the most frequent of the sympathetic affections, but in which auscultation furnishes only negative results.

The lithic diathesis, irritability of the bladder betrayed by frequent micturition, scantiness and scalding in the passage of the urine, were among the rare and occasional symptoms—high-coloured urine was probably oftener present than noticed.

The encephalic organs were a common seat of morbid phenomena—the most frequent of which was headache; excluding still those cases in which the nervous centres with other systems had become more seriously and permanently interested in the general disorder of the health. Sometimes this was the most urgent complaint: when attended with vomiting, it evidently was immediately dependent on the actual state of the stomach at the moment; at other times, by its intensity, duration, and its being accompanied by other cerebral symptoms, some fears were entertained that the brain might be the seat of the primary disorder and the stomach only secondarily affected, in which case the result alone could correct the diagnosis. Next in order of frequency, among the sympathetic cerebral symptoms, was vertigo; and occasionally there was a sense of weight in the head, especially in the occiput, with drowsiness or a distressing noise in the ears. Among nervous symptoms not confined in their seat to the head, were heaviness of the limbs, aching of the legs, and occasionally numbness of one side or in the præcordial region. In one instance a neuralgic affection of the 5th pair preceded the debut of the gastric disorder—a loss of strength or muscular power was early a very common complaint, and also one much insisted upon by the patients even after the urgency of the gastric symptoms had been removed. Low spirits, mental disquietude respecting their malady, persuasion of benefit to be derived from particular remedies, increased susceptibility to moral impressions, might also be enumerated among the occasional sympathetic affections. Various anomalous sensations, which would often fasten on the notice of the patient, it would be useless to dilate upon.

Such were the most ordinary symptoms which occurred as the accompaniments of the gastric affection—the reverberation rather of the central disorder in distant parts of the system than its actual propagation to these parts—and thus far a description merely has been given of the most ordinary of all complaints, familiar in its features and history to every practitioner, easily recognized in every case, at once pronounced to be dyspepsia, and dismissed accordingly with some favorite formula of prescription ready at hand for such patients—and indeed little excuse could be found for occupying the pages of a periodical with a communication of so little novelty, where no claim is made to any therapeutic discovery, beyond its title to be a sketch from the life, or an analytical reduction of cases to a general statement; and in a statistic respect to its being a representation of the frequent occurrence of a form of disorder—more commonly insisted upon as the compensating penalty for the possession and use of the luxuries of life and abundance—among a class of people whose social condition precludes the supposition of such an origin of the disorder, suggesting rather one of an opposite character. But such an exposition of this common form of disorder would not have been introduced except for the ulterior purpose of showing that it was the germ, the latent and abiding cause of a series of morbid changes, which produce extensive havoc in the constitution; and that its frequency and persistance and its consecutive evils maintain no inconsiderable portion of the lower orders in this district, at least the female part, in a state of prolonged ill health, and constitute a heavy tax on the funds provided for their support.

### *Predisposing Causes of the Gastric Affection; and circumstances and conditions modifying its nature and consequences.*

It was chiefly however from being enabled to trace in the records of the cases, forming the subject of this communication, notices of such circumstances as seem to have been the predisposing or sustaining causes of the disorder, that these preliminary remarks have been offered on the form of the gastric affection itself and the attendant or consecutive derangement of other organs or functions. So large a proportion of the cases of a provincial hospital being of the above description, and no inconsiderable amount of the sufferings of the class of persons seeking hospital relief being derived from this source; it would have been scarcely sufficient in a clinical report to have stated merely, that the prevailing disorder was that of functional disturbance and morbid sensibility of the stomach, producing more or less frequently, serious derangement of the general health ; especially when the purpose was to show, that there must exist something in the social condition or habits of this class, to account for the great preponderance among them of a particular form or family of disorders. And such a previous history seemed the more called for, inasmuch as in most of the treatises on dyspepsia, this malady is represented more especially as the consequence of modes of living from which the greater number of the subjects of the present cases were excluded by their position and circumstances : for, if in some of the present cases, the causes of functional disorder of the stomach were referrible to abuse of its functions or to excess, as in a majority of the instances in other ranks of life ; in not a few of them the opposite condition of insufficient nourishment might be assigned as the predisposing cause ; but in by far the greater proportion, circumstances, interesting the whole organization but interfering especially with the due performance of the gastric functions, without any directly noxious cause acting on the stomach itself, originated and maintained the disorder.

While therefore the gastric affection, such as it has been described, remained the same for all the cases, and the general mischief to the system was such as has been delineated, the cases admit of being further reviewed under three subdivisions, according to the predisposing conditions of the system or external producing causes, to which it seemed reasonable to attach the disorder. It may be premised that, as more than nine-tenths of the cases were of females, the causes, whatever they may have been, must have had a peculiar relation to the physical or social condition of the sex.

*Abuse of the Powers of the Stomach.*—In the first subdivision then, those cases are included which were referrible to abuse of the powers of the stomach, including the ordinary causes of indigestion ; as intemperance or the use of unwholesome food, and, not unfrequently an abuse of the stomach of a different nature, viz. the habit of overdosing with domestic medicine. The first, as might be expected, was most common among the male, and the latter among the female patients. As the cases of this subdivision presented nothing peculiar, they need not be insisted on ; a short notice may however be here introduced of one instance, for the purpose of showing how intolerant the stomach may be, even when itself not the seat of any particular disorder, of an ordinary article of food, where the general state of the system in chronic maladies renders ordinary diet inappropriate. An hospital patient had for months been confined to the horizontal posture for a severe and tedious disease affecting the osseous structure of the pelvis, but from which he was slowly and unexpectedly recovering, when one day he was attacked with erysipelas of the lower extremity, and, the day after the febrile accession which ushered in its attack, he was seized with vomiting, and rejected from the stomach an oblong white concrete substance, about three or four inches long and nearly an inch broad, which on examination proved to be a mass of cheese kneaded together by the motions of the stomach : cheese not forming an article of the hospital diet, it was found on enquiry, that ten or twelve days previously he had eaten some cheese brought to him by a friend, and none in the interval—the erysipelas soon disappeared after this accident.

The practice of taking purgative medicine in every indisposition, slight or serious, previously to taking medical advice, has become the prevailing habit among the females of the laboring classes and domestic servants, and is almost regarded by them as one of the necessaries of life. Epsom salts, taken in doses of an ounce at the time, being the drug almost universally employed. This domestic remedy with perhaps the scarcely less injudicious drugging administered by unlicensed practitioners, the author is persuaded is a frequent cause of the disorder under review, among persons applying for hospital relief; and in not a few of the present instances it has been possible to trace it to this source. It would be quite needless to insist here upon the pernicious tendency of such a habit, and of its peculiar liability to weaken and derange the powers of the stomach. It is to be lamented that the misdirected benevolence of the better-informed should increase the evil by furnishing similar means for undermining the healths of the applicants to them for relief.

*Insufficient or Innutritious Food.*—A second subdivision of the cases admits of being formed, in which food, insufficient in quantity or deficient in nutritious quality, considering the degree of exertion or exhaustion entailed on the patients by the nature of their occupations, might be assigned as a predisposing cause.

Although few if any of the cases could be said to be solely attributable in their immediate origin to impoverishment of diet, yet investigation into the circumstances of many of the patients rendered it highly probable that this cause existed among others, and, with the concurrence of other circumstances, mainly contributed to the production and continuance of the disorder.

As the object of these Contributions embraces the statistical relations of the subjects treated of, it may not perhaps be irrelevant to introduce here a brief statement, to show how the labouring classes in this district are circumstanced in respect to diet, although there may not exist much difference in this point from what is the case in other parts of the country. It may be remarked then, that the families of the poor resident in the town of Cambridge are so far differently circumstanced, if not from the population of other towns at least from the surrounding agricultural population, that they are enabled to procure good animal food at a very reasonable rate, so as most frequently to have a dinner of butcher's meat three times a week; whereas the families of the poor in the villages scarcely ever procure any animal food except bacon, and that but rarely, so as to furnish little more than one meal in the week. This distinction arises from a circumstance peculiar to the town of Cambridge, in that for the greater portion of the year, during which the members of the University are in residence, large quantities of broken victuals, the perquisite of college servants, are sold at comparatively a very cheap rate. In contrast with this condition of the townspeople, a statement is subjoined of the quantities and nature of the articles of food consumed, taken indifferently from different villages, in the county.

A family, consisting of man and wife with seven children, 15s. a week, being earned by the father and eldest son.—Weekly consumption. Three stone of flour, three pecks of potatoes, 1½lb. of cheese, 1½lb. of pork, ½lb. of butter, 1½ oz. of tea, 1lb. of sugar.

A man and his wife with four children earning 9s. a week.—Weekly consumption. Three pecks of brown bread, ½lb. of butter, ½lb. of sugar, and ½ oz. of tea. The husband has his breakfast in the fields, consisting of some milk of his master's with his own bread—at dinner has some small beer of his master's—less than once a week a little broth or milk for the family from the minister.

A man and his wife with one grand-child, husband earning 8s. a week.—For breakfast the husband has a mess of water-gruel with bread, the wife a little tea with bread. Dinner, pork with potatoes and bread, no beer—for the evening meal, the wife has tea with bread and butter, and the husband bread and cheese with tea—the child a "mess with water out of the kettle"* for its meals. Weekly consumption, 1½ stone of flour, 2lb. of pork, ¼lb. of butter, ¼lb. of cheese, potatoes from their own garden—1 oz. of tea.

---

* Boiling water poured over bread, with sugar added, and sometimes dripping given them by the farmer, is their ordinary mess; their gruel is made of wheaten flour and water.

Husband and wife and one child,—Weekly consumption, 1 peck of flour, ½ peck of potatoes, ½lb. of cheese, ½ or ¾lb. of butter, ¾lb. of sugar, 1 oz. of tea, 2 oz. of coffee—about twice a week pork for the husband only.

It is not here insinuated that this almost total absence of animal food contributes to render the agricultural population more ailing than it otherwise would be, any further than that it does not provide for the contingencies of original delicacy of constitution, trying circumstances from critical periods of life, severe labours and other predisposing causes of the disorder, to be considered in the next sub-division, and thereby contributes to retain the less robust portion of the population more frequently and longer among the ranks of the sick than if their circumstances admitted of greater latitude in the adaptation of food to pressing exigencies. It is not within the scope of the present subject to consider whether it would at present be judicious or practicable to make a diversion of a portion of the funds provided by benevolence or otherwise for the poor to this purpose; but it is certain that it is both a waste and a mockery to be liberal of the resources of pharmacy when no means are devised for better provisioning the cupboards of the poor. These remarks apply almost exclusively to the laborious yet feeble mothers of families residing in country villages, who constituted a large portion of the subjects of the present cases.

*Conditions of the System Generally, Interfering with the Gastric Functions.*—In the third subdivision those cases are included in which, without either abuse of the powers of the stomach and independently of any insufficiency of diet, the conditions of the system generally from internal or extrinsic causes were unfavourable to the due performance of the functions of the stomach, and therefore originated disorder of that organ. These conditions were various, of which the following may be adduced as the principal.

1. Original delicacy of constitution. To those placed under domestic circumstances requiring the same exertions with others possessing ordinary stamina and yet not having the means of more generous living, especially if mothers of families, this constitutional delicacy is a severe trial, frequently entailing on them that atony and trouble of the digestive functions which is matured into a confirmed state of gastric disorder; on enquiring of such persons the origin of their complaints, the general reply is that they have been ailing for years, and yet they present no signs of organic disease; such become frequent pensioners on the hospital, returning to be cured of their "old complaint," and their appearance bespeaks them aged beyond their years.

2. Particular epochs of life—as the debut of puberty or the constitutional effort towards the establishment of the catamenia—and the climacteric period. At both these epochs, the habits and ordinary hardships of females of the poorer classes tell upon the constitution with more than ordinary force, and the stomach seems the first to resent the ill-conditioned circumstances to which the entire organization is subjected.

With respect to the former of these epochs, its usual coincidence with the period of first going to service, the exchange at that critical time of the country air for confinement to the house in a town and of the lighter occupation of aiding a mother in her domestic duties, to the greater and more continuous efforts of a "maid of all-work," may be assigned as causes which prejudice the assimilating powers of the gastro-intestinal organs, at a time when their integrity and vigor are more especially required to meet this new effort and demand of the constitution: a sixth nearly of the cases, domestic servants living in a town, were of this description. The change of diet, although for the better, proves by no means the compensating advantage it might otherwise be, were it not for other changes more inimical to the economy: indeed such alteration of diet may rather prove prejudicial by unseasonably imposing a new habit on the stomach. The gastric disorder presented in these, for the most part, the same features as in the other classes of patients, but was oftener attended with vomiting; and, whereas, with the rest, constipation was much more frequently the habit of the bowels than diarrhœa, with them the latter condition was as commonly met with as the former. The resulting functional derangement of other organs and systems was the same, and presented the same varieties as appertained indifferently to all exhibiting that condition of the economy which has latterly acquired the name of anæmia, and which will be considered more at length hereafter. It may be remarked, however, that not only was the elaboration of the catamenia, under the unfavourable circumstances above insisted on, rendered effete and difficult, but that the effort itself seemed often to have been postponed to a later period from the same causes, and especially precocious labor.

The second, the climactric epoch, marked by the decline of the catamenial function, seems also especially favorable to the occurrence of the disorder: about one third of the cases of married females affected with the gastric affection being so circumstanced—with these likewise, as with the last mentioned, it was characterized by irritability as well as morbid sensibility of the gastro-intestinal canal; retching and vomiting being more frequent with them, and diarrhœa oftener taking place of the more ordinary condition, constipation. With respect also to the general derangement, not uncommonly its features during this decline of the catamenia very much resembled those which characterized the disorder when incident during the immaturity of the same function; and especially as regards phenomena denoting the implication of the circulating system and which have already been delineated. Unfortunately the resemblance does not extend to a corresponding susceptibility of alleviation from remedies; a difference very conceivable, since at the later period when the arterial system has lost its pliancy any disturbance of the circulating powers must present a much nearer approach or affinity to structural alteration than at the early age when the vascular system however deficient in tone retains its physical integrity.

3. Another by no means infrequent predisposing cause of the malady among the same class of persons, although not incident to many of the present cases, is undue lactation; *i. e.* lactation maintained under unseasonable circumstances as to health or constitution, or oftener, protracted beyond a seasonable term: from one and a half year to two years is not an uncommon period among these persons to continue nursing. The stomach, however inadequate for a period to perform its part in elaborating nourishment beyond the wants of the individual economy, is the first to "strike" when this imposition of extra-duty is too long demanded of it: it is not surprizing therefore that among such females "dyspepsia lactantis" so frequently occurs. The sinking at the pit of the stomach is with them the most prominent symptom, but most of the phenomena already described are liable to be associated with it: though the instances that have occurred to the author have not exhibited that extreme disturbance of the system which has occasionally arisen under the agency of other predisposing causes.

4. Another circumstance, which from a review of the cases, appeared obviously to have been the origin of the complaint in several, was the precedence of some acute disease or of some exhausting accident, as abortion. The mode, by which such antecedents predisposed to the gastric disorder as a sequelæ, was by a premature return to the usual avocations and exertions during incomplete convalescence. Acute diseases are more commonly converted into chronic affections of the like nature, among the poorer classes on this same account: but without this result ensuing, the health is liable to continue impaired or insecure, from time not being allowed to the stomach to be properly reinstated in its powers; since the reparation of the exhausted system is a sufficient tax upon its energies, even in repose: and this it may be inadequate to, if it has at the same time to meet the demands of renewed activity in the muscular system. In several instances the disorder was traced to convalescence from scarlet fever and acute rheumatism; nothing however is more frequent than for hospital patients to complain that they have never "had their healths" since they were subjects of such and such acute malady—and it will be found that a large proportion of patients making this complaint are sufferers from gastric derangement of the kind under consideration ; too early dismissal of patients from the wards of an hospital, or the making out-patients of convalescents, thus often becomes the means of draining the funds of the institution by converting them into pensioners on its resources.

5. It might be expected that the presence of some morbid diathesis, as that of scrofula or phthisis, should have in several of the cases been coincident with the gastric disorder, and indeed, in some instances, the gradual development followed by the advanced stages of phthisis was observed to ensue upon the functional derangement of the stomach. Without insisting too much upon the presence of such a diathesis as a predisposing cause of gastric disorder, it appears certain that the stomach is often the earliest to betray the existence of some "fault" in the constitution. In several of the present cases tuberculation of the lungs seemed to be imminent, when not yet declared by any certain physical sign.

Under this head may be mentioned another, the aguish diathesis, if it may be so termed, as being in some way connected with the occurrence of the gastric affection. In some cases ague had simply preceded it, but in others no regular intermittent was at any time formed in the course of the affection, but distinct paroxysms, simulating ague, would occur at irregular intervals ; occasionally again, where no actual exposure to malaria could be supposed to have occurred, ague would at an advanced period of the gastric disorder for the first time make its appearance. In all these instances probably the patients came from aguish districts or had been the subjects of ague at some former period of their lives ; is it possible, however, that the gastric disorder may sometimes be a latent form of intermittent?

6. As in other disorders, so likewise in this, the habit of the disease itself sometimes acts as a predisposing cause of its recurrence ; at least in some of the cases the statements of the patients seemed to indicate this, viz. alleged returns of the complaint at various periods without any obvious exciting cause, and with intervals of ordinary health. It is to be noted, however, that such is the early history of persons in whom organic disease of the stomach is subsequently developed.

7. A state of the system still remains to be considered, which in by far a greater proportion of the cases, *i, e.* 40 out of a 101, occurred as the predisposing cause of the primitive gastric disorder. It was that state in which, in the progress of the complaint, those characters, and combinations of symptoms were presented, which collectively have acquired the nosological distinction of anæmia, chlorosis, and frequently the more exclusive one of amenorrhœa. This section of cases has been reserved for the last, inasmuch as to substantiate their claim to be comprehended among the other instances of gastric disorder, required something more than their bare enumeration. The cases referred to, (exclusive of those already considered when treating of commencing puberty as a predisposing cause, and with which they presented the greatest similarity) were all of unmarried females under the age of 30, 28 out of the 40 were resident in towns and three fourths were domestic servants, and of the remainder, several were milliner's apprentices; in all these there existed, in quite as much prominence as in the other instances, the symptoms of gastric disturbance that have been detailed in the foregoing pages, and which, abstracted from their complexional characters, constituting the title-page of the so-called disorders, anæmia, chlorosis, &c., would still have entitled them in any arrangement to be classed with gastric affections, and to have passed current as dyspeptics ; and in every case nearly the first notice of returning health was the improvement in the gastric functions. With regard to any peculiarity in the gastric or intestinal disturbance distinguishing them from the rest, little is to be remarked ; retching and vomiting were much less frequent; pain in the left hypochondrium perhaps more common, while constipation in degrees varying from torpor of the bowels to obstinate costiveness, frequent among all classes of the patients, was especially so among these; but that this condition was dependant on the gastric affection and not its cause was very clear, from its seldom yielding to mere purgative remedies. It would be a waste of space to enter into much detail of the symptoms of the general disorder, as they are at present so well understood, and have been so accurately detailed by Dr. Marshall Hall. It may be noticed, however, that these cases without prejudice to the identity in all of the gastric disorder, and while retaining a marked resemblance to one another in the general derangement, presented three varieties, accordingly as the disturbance in the circulating, respiratory, or cerebro-spinal functions predominated. To the derangement of the functions of circulation, indeed the physiognomy of the entire disease in all, might with propriety be referred; since to this appertained that group of symptoms from which its name, anæmia, was borrowed—as the pallor of the countenance and lips, dark circle round the eyes, the exsanguine appearance of the tongue, gums, and fauces, &c.—conditions depending upon lesion of the capillary functions or the composition of the blood itself; but if these symptoms were more or less common to the whole section of cases, as well as more or less disturbance at the centre of the circulation, as indicated by palpitation on slight exertion, or on first lying down, and habitual acceleration of the pulse. In others the disorder of the circulation was so predominant as to mask the affection of other organs: extreme and distressing palpitation, anormal sounds of the heart, as a bellows-murmur accompanying the first sound, or a sharp and clangorous quality of the second; throbbing of the arteries of the head or of the whole frame; a vibrating, thrilling, or even irregular pulse, frequently full without being firm; in other cases faintishness or actual syncope; more or fewer of these phenomena and in various combinations constituted in many the most urgent and pressing part of the malady, and the palpitation perhaps almost solely engaged the attention of the patient, while in the instances in which there was not this predominance of disorder in the functions of the circulation, the complaint of palpitation was only elicited by enquiry. The bellows-murmur was often the last to disappear of this group of phenomena interesting the apparatus of the circulation. It is such cases as these that are quoted as instances of nervous palpitation, while in fact they still repose upon the gastric disorder as the initiative and sustaining lesion. As an appendage to the derangement of the circulation, an anasarcous diathesis was observable in many, in various degrees, from slight leucophlegmatia of the face and infiltration of the ancles, which was very common, to positive œdema of so considerable extent as when dropsy was regarded as an idiopathic affection would in them have constituted a disease of itself.

The second variety, in which the respiration seemed most prominently implicated in the general disorder, did not include many of the severer cases. Dyspnœa and cough, generally unaccompanied by expectoration, were very frequent; but in some these formed the principal symptoms of which the patients themselves complained: yet it was by no means uniformly, in these instances, that a closer investigation elicited indications of incipient or imminent phthisis; as where it was found that slight hæmoptysis had at some period occurred, or in which auscultation detected feebleness of the respiratory murmur in one or both clavicular regions. The dyspnœa and cough, thus pressed upon the notice of the patient herself, for the most part seemed to depend upon the functional disorder of the heart and blood-vessels which accompanied them.

Phthisis, however, did originate in some of the cases during the gastric affection, and was suspected to be latent in several. Hoarseness, partial aphonia, or alteration in the tone of the voice, were occasionally incidental to the disease; but these probably belonged essentially to the group rather of nervous symptoms than to those proper to the respiratory organs.

The third variety, in which there was a predominance of affections of the head, principally, included a considerable proportion of the cases. Head-ache, indeed, was nearly a constant complaint, and vertigo almost as much so: but the intensity and persistance of the former of these symptoms, occasionally, was such as to be the sole complaint made by the patient; and such cases might with propriety have been designated as cephalalgia, if they had not been associated with the gastric affection common to all. In general, these symptoms seemed to depend upon the actual state of the cerebral circulation, as they were most frequent on first rising in the morning, on exchanging, therefore, suddenly the recumbent for the erect posture; sometimes, but rarely, there appeared to be a state of passive congestion of the brain, as indicated by continued drowsiness. With this class of patients likewise, as well as with the others, but not more frequently, there occurred various other symptoms appertaining to the cerebro-spinal system; but which do not merit particular notice. Cramp in the legs was not uncommon, connected seemingly with flatulence of the large intestines. Loss of strength, and tremors of the tongue and lips, belong to the share the muscular system had in the general impairment of the health. The state of the tongue, as regards flabbiness, indented margins, creased surface, &c. which has been so much insisted upon by Dr. M. Hall, may be mentioned likewise, in connexion with the last-named symptoms; it may be stated, however, that the observation of these different conditions of the tongue, did not confirm the remarks of the author just quoted, relative to their degree and varieties corresponding to the intensity and duration of the general disorder of the health

It remains to mention the participation of the catamenial function in the general derangement in the cases under immediate consideration—a function, the disturbance of which, in such cases as these, of anæmia or chlorosis, would undoubtedly, at no distant period of the art, have been pronounced as the *origo mali* whenever it occurred. Indeed, with very few exceptions, there was uniformly more or less irregularity of the catamenia: positive amenorrhœa was, perhaps, not more frequent than among the rest who did not present the features of the general disorder just detailed; but scantiness, discolouration and irregularity of the menstrual discharge, was almost in every instance acknowledged by the patients. That this impairment of the catamenial function was but one of the consequences, one, indeed, of the most remote, of the primitive impairment of the digestive functions, was sufficiently evident; inasmuch as it was in general the last that was restored to its normal state, and the patient would be discharged apparently in the full enjoyment of re-established health, while this part of the economy was still at fault; or its renovation would result as the tardy climax in the series of reinstated functions.*

Having thus completed the sketch of the train of symptoms characterizing this subdivision of cases, the question remains to be solved—if in these likewise the derangement of the gastric functions be admitted as the original and abiding malady, and the source of the ulterior changes in the system—what were the conditions which determined, in these particular instances, the extension of the gastric disorder to take this direction, and to issue in these characteristic lesions of various functions and systems? The class of persons affected, seems to point

* One of the cases forming the subject of the present communication may be here alluded to, as having presented a remarkable idiosyncrasy of the menstrual function, but not singular in the records of medicine. It was of a female, aged about 38, who, during her married life, averred that she never menstruated except when she was pregnant, and had never menstruated since she had been a widow.

readily to the solution, viz. domestic servants living in towns, and almost entirely confined to the house; or needle-women, still more rare indulged with breathing the open air; with the aggravating circumstance of habitual sedentariness and confinement of posture—in addition to which, a large proportion of the individuals had not arrived at the period of full maturity of conformation, or such completion had been retarded or postponed beyond the proper term. These circumstances seem to denote that if they were prejudicial to the due performance of the assimilating process where it begins in the stomach, the effects of this initiative derangement must have been peculiarly liable to be propagated, so as to disturb and pervert the whole circle of assimilating and nutritive functions, and especially that of sanguification, by the absence of those conditions of the capillary system of the lungs and integuments, in its relations with the external atmosphere, which both theory and observation show to be most essential to the health of the economy. In other words, the physical circumstances in which these persons were placed were equally prejudicial to the commencement of the assimilating process in the stomach, and its completion in the lungs.

That other circumstances conspired with this predisposing cause, viewed as the principal, is highly probable, when the age of the patients is considered; and they might be such, as bearing on the social habits and moral condition of this class of persons, do not come within the compass of clinical observation to discover or notice.

*Treatment.*—Thus far then, from the foregoing analysis of cases if correctly represented, it would follow, that functional disorder of the stomach occurs in hospital patients, by no means in the simple form of dyspepsia merely, but as the basis and sustaining cause of a variety of morbid states, which have in general been regarded and treated as separate disorders. To the unity of the gastric affection amidst various complications resulting from the different circumstances under which this primitive disorder originated or was maintained, the attention has been principally invited, inasmuch as it appears most essential, that, in the treatment, this should be steadily kept in view, as furnishing the only rational indication for the choice of remedies and for perseverance in their use. The comportment of the gastric affection under any particular mode of treatment being the test of its general usefulness in the restoration of the entire health in every instance. A short statement then of the effects of the remedies employed will terminate this analysis of cases.

Although such cases as the present furnish little that is interesting in the clinical annals of an hospital and may be somewhat irksome from the frequency of their occurrence and their similarity; yet the degree of success attending their management forms a very considerable item in the general amount of service rendered by the institution, and very materially concerns its credit and economy. Unfortunately their permanent cure depends, more perhaps than in any other disorder, on circumstances of regimen, over which the hospital practitioner has a very limited or only a brief control; hence it is, that these patients become the most common pensioners on the funds of public infirmaries: hence also, of the present cases, those treated in the wards recovered much more speedily and entirely, than those who attended only as out-patients, who had not the means or opportunity, if they had the inclination, to adhere to proper regimen or diet. In some few instances, indeed, the abandonment of previous errors of regimen was alone adequate to produce prompt recovery, without any aid from medicinal remedies. It is not intended, however, to enter upon the subject of the dietetic and regiminal treatment of the cases; but merely to state, that, with the exception of excluding the use of green or fresh vegetables, the choice of animal or farinaceous aliment in each instance was determined, accordingly as the stomach appeared best to tolerate one or the other. With the mothers of families, when subjects of this disorder, little could be effected beyond an alleviation of symptoms, when their circumstances did not admit of their procuring an increase of domestic comforts, and repose from their ordinary labours. One instance, however, may be quoted, in which a remedy of an opposite nature was found most effectual; the disorder which had long tormented the patient, being speedily terminated by the invigorating influence of gleaning. With domestic servants, who numbered at least the moiety of the cases, the treatment was abridged or prolonged, accordingly as they were induced or not to forego service for a time, or in proportion as their employers allowed them the licence of exercise in the open air. If the treatment of the disorder should ever engage more than the passing notice of the practitioner, and the necessity of entertaining more enlarged views of its importance in respect to the statistics of medicine, and in its relations to the hygienic state of the community, should be acknowledged; it is certain that any effective control over its extension must depend upon the practicability of improving the habits and condition of the classes chiefly affected by it.

But to proceed to the consideration of medicinal remedies—and, in the first place, of those which are generally regarded as acting immediately on the functions of the stomach—especially bitter infusions—these were never much relied on, and but rarely exhibited, except in combination; and indeed to prescribe them with a view to exalt the appetite of those, who, at their own homes had no means of procuring suitable aliment, would have been of questionable utility: with the addition of alkalies, in a few instances only, did they appear of service, where flatulence of the stomach, with or without acidity, prevailed. Of the effects of other remedies, especially hydrocyanic acid and bismuth, the notes of the cases bear more favourable testimony; not as more adequate to their cure, but as controlling very materially the morbid sensibility of the stomach. In the cases in which the acid seemed to be most serviceable, acute pain in the epigastrium during digestion, or craving during fasting, were among the most distressing of the gastric symptoms, and which often yielded promptly to the acid, taken at the instant of greatest suffering, in doses varying from ℳj.—ℳiij. of Scheele's acid, and never exceeding three doses in the day: the craving sensation of hunger was sometimes immediately suspended on taking a dose of the acid. In one case, in which the extremely cachectic state of the patient indicated severe inroads on the constitution, the acid was still found a great resource in controlling the epigastric pain: and, occasionally, when sinking at the pit of the stomach was the residuary complaint, after graver symptoms had yielded, this likewise was relieved by the same remedy.

Bismuth seemed to be useful under much the same circumstances as the acid; nor was much discrimination used in the choice between the two; perhaps the bismuth was more efficacious, where, with the epigastric pain or craving, there was flatulence, and perhaps also it had a more direct influence in improving the appetite when deficient. In no instance did it seem injurious, or to cause any unpleasant symptoms.

Both these remedies seem deserving of trial, as capable of affording relief in the numerous cases of gastric disorder, associated with organic disease of neighbouring organs, especially the liver. Hyoscyamus was another medicine, of the class acting on the sensibility of the stomach, which was exhibited; but chiefly in conjunction with minute doses of blue-pill and aloes; such a combination seeming to interfere least with the state of that organ, when it was the object to act gently on the intestinal canal.

Local applications to the epigastrium, as the tartar-emetic ointment or blisters, may, from their observed effects, be enumerated among the remedies controlling the morbid sensibility of the stomach: they were seldom employed in the cases presenting the condition of anæmia, and upon the whole were not to be reckoned among the most serviceable of the remedies used. Leeching the region of the stomach seemed in no instance to be required. It may be here incidentally mentioned, that one of the most severe cases, marked by extreme irritability of the stomach and disturbance of the circulation, occurred after free venesection had been injudiciously employed for a supposed inflammatory condition of the abdominal viscera.

Passing from the acknowledged gastric remedies, the next remedies to be noticed are those, which, though generally distinguished as tonics, and vaguely supposed to act by giving tone to the solids of the body or by improving the qualities of the blood, from the observation of the present cases, seem entitled to be regarded as acting primarily on the functions of the stomach; as they were found serviceable in the general improvement of the health, only accordingly as, in the first instance, their exhibition was attended with increased energy in these functions and abatement of the distress attending the process of digestion; the remedies alluded to, are principally the preparations of iron. The cases in which the employment of this remedy was most efficacious, were, as might be expected, those distinguished by the presence of anæmia: and certainly no pretension is made to any novelty of practice in this respect, except it be in the extent to which it was carried; it was almost universally used in this division of cases, and in several of them was the only remedy employed. The preparation most frequently used, was that recommended by Merat and De Lens in their Dict. de Matière Médicale, in which carbonate of potash and sulphate of iron in equal quantities are decomposed by simple trituration; it was given in the form of pills or electuary, in daily quantities varying from ʒj. to ʒiss. This preparation was found far more efficacious than any other, probably from the decomposition and conversion into a sub-carbonate of iron being more complete; very rarely, indeed, was it found necessary to suspend its use from its producing any disagreeable effects. The head-ache, which was almost uniformly present in these cases, was no bar to its use: the earliest admission, on the part of the patient, of improvement after begining this treatment, was usually conveyed in the expression, that they could "eat better:" all attempts to lessen the disorder by improving the diet often failed, and the stomach would rebel against it, till the chalybeate treatment had been employed some days. The use of iron, however, was not confined to this section of cases, but was found of service in many others; but in these latter instances it was in combination mostly with aloes and in the form of the sulphate that it was employed; three weeks or a month was the ordinary duration of the treatment in those cases in which iron was the remedy principally relied on. Next to the improvement in the gastric functions, one of the earliest indications of the good effect of the remedy, was a marked improvement in the complexion; at a later period abatement of the palpitation ensued. When the chalybeate did not prove beneficial at the commencement of the treatment, and usually when active purging was required, it was still frequently found to hasten and complete the convalescence when employed at a later period. Sulphate of quinine was occasionally used in combination with iron or alone under the same circumstances and with similar but less striking results.

Next to remedies addressed more especially to the stomach, those acting on the remainder of the canal naturally occur to be noticed. In the same proportion of cases and in the same degree as constipation of the bowels was present, recourse was had to the use of laxatives or purgatives, much more frequently as merely an auxiliary and occasional part of the treatment, than constituting its most essential department. A combination of aloes, rhubarb, and Rochelle salt, given once in the morning, was the laxative most commonly employed, when the object was simply to assist in procuring a daily evacuation; and the resinous purgatives in larger quantities with the addition of sulphate of iron were used, when more rigorous purging seemed requisite.

It was rare that much advantage was derived from the use of mercurials, even in alterative doses; mercury was never employed with benefit in the large proportion of cases characterized by anæmia, but was chiefly serviceable where the tongue was more than commonly furred, and where vomiting or retching were present, or the secretions of the bowels disordered. In these instances the blue-pill in doses of gr. ss.—gr. j. with *extr. alo. and extr. hyosc.* ā ā, gr. j., or with *pulv. calumb.* gr. iij. *ter quotidie,* was the most usual mode of exhibition.

Distressing symptoms affecting other organs and their functional disorders for the most part failed of being relieved by direct remedies and but ensued on the amelioration of the gastric affection. This was especially the case with the group of symptoms already described as interesting the circulation, especially the palpitation and dyspnœa: belladonna applied as a plaister to the cardiac region seemed but in a single instance to have been of service. In one case incomplete aphonia, which did not yield to external applications to the region of the larynx, ceased speedily after commencing the treatment by preparations of iron. Rather more latitude was practicable in alleviating the affections of the head: even in a generally asthenic state, cupping the occiput was found sometimes of service, in removing head-ache when accompanied with vertigo and drowsiness; and in one instance already quoted, vomiting, which had long existed, ceased to recur after the use of this remedy: but the most frequent relief to the ordinary head-ache was derived from the application of blisters behind the ears; very rarely this troublesome accompaniment yielded to valerian when other means had failed.

As the general result of the 101 cases it may be stated that of these, 62 were discharged recovered, 25 benefited, and 14 discontinued attendance without having received any relief. Those cases in which the chalybeate treatment was found admissible, were by far the most satisfactory in the immediate result, but relapses were not uncommon among them at no distant period from the date of the first discharge. The development of phthisis, during the treatment did not interfere always with the recovery from the gastric disorder.

*Second Division of Gastric Affections, or Inflammatory and Organic Diseases of the Stomach.*

This second division of gastric affections includes the cases in which it was more or less clearly denoted that there was an excess of blood congested in, or circulating through, the coats of the stomach.

*Inflammatory Dyspepsia.*—It has already been stated that the reference of cases to this division was often determined rather by the nature of the treatment found successful than by the presence of any pathognomonic symptoms—and it may now be added, that, intimately allied with the cases of the foregoing division in the general features of the gastric affection above detailed, and excluding the occasional presence of none of its symptoms, there occurred a number of other cases, which have been separated from the former upon no other absolute grounds, than the different nature of the remedies that were found efficacious, and which, in one word, were antiphlogistic: this perhaps may entitle them to be distinguished as cases of inflammatory dyspepsia; premising that by inflammatory is intended a tendency to, rather than the actual presence of, inflammation. But although these cases presented no symptoms, which were not occasionally met with among the former; still the greater intensity or comparatively more frequent occurrence of some of these symptoms or conditions may be mentioned, as coinciding with the more absolute contrast in the treatment, to justify a separate analysis of them. Combining then the results of the treatment with this greater frequency or predominance of certain symptoms, the following conditions may be assigned, as the principal grounds, on which they were considered as cases of inflammatory dyspepsia, and therefore distinguishable from those of the foregoing division: these conditions or results are placed in an order corresponding to the importance attached to them in a diagnostic point of view, commencing with the most important.

1. Relief experienced from blood-letting, especially by leeches to the epigastrium or its vicinity.
2. The good effects of rigid abstinence in aid of other remedies.
3. Redness, marginal or terminal, of the tongue, with more or less dryness.*
4. The presence of pyrexia of more or less intensity.
5. Urgent thirst.
6. Sensibility of epigastrium to pressure, amounting to tenderness.
7. Bitter taste in mouth.
8. Vomiting.

More or fewer of the above conditions or results were observed in each of the cases at present under consideration; and, the higher in the list the more frequent was their occurrence. This form of gastric disorder is far from being so common in the practice of the hospital as the former: indeed, during the same period that notes of the 101 of the former description were taken, only 12 of the latter have been recorded: considering their paucity, therefore, no formal analysis of them is admissible. These likewise, were all cases of females, but on the other hand they differed from the previous cases, in the subjects being generally robust, and occasionally, if not for the gastric yet for incidental affections, they required and were benefited by general blood-letting. In two of the cases there seemed to be a remarkable aptitude to inflammatory action and irritation of the mucous membranes; an erythematous inflammation of the fauces occurring once or oftener in the course of the gastric disorder; at another time, a smart attack of diarrhœa; in one of the two cases, erysipelas of the face followed the gastric affection, but was cut short by blood-letting; in the other, a young unmarried female, was subject at the same time to severe menorrhagia. Of the gastric affection little remains to be added, beyond what has already been stated for the purpose of contrast with the former description of cases. In one instance the inflammatory symptoms seem to have only supervened after long-continued retching; in this case the attack was brought on by mental anxiety and yielded in a great measure to repose.

In another instance, attended with distressing thirst, cold drinks of any kind greatly aggravated the pain at the epigastrium.

With the exception, that in none of the present cases was there that general state of the system, to which the term anæmia has been appropriated, other organs and functions seemed to be sympathetically or by extension of disease implicated, much in the same manner and with the same varieties as in the former division of cases. It is to be observed also, that, in several, there seemed a disposition in the disorder, on the subsidence of the inflammatory symptoms, to pass into that form characterised merely by impaired function and morbid sensibility; and a general asthenic state to replace the inflammatory diathesis.

---

* As a singular exception to the general rule, that redness of the tongue indicates an inflammatory condition of the stomach; the case of a young female may be here noticed, who is a frequent applicant to the hospital for relief from a disorder she is continually subject to, consisting in a constant and most distressing pain in some region of the spine, with aching and occasional numbness of the lower extremities. The cervical region and the occiput are the most frequent seats of the pain. In the interval of her attacks she appears tolerably robust, but, after a severe and prolonged attack, she has presented a state bordering on anæmia. There are seldom any direct symptoms denoting any gastric disturbance; but the singularity of her case consists in the tongue, during these attacks, being quite dry down the centre and of a bright red, without there being any local cause or the presence of fever to account for it. Leeches to the epigastrium had frequently been applied without being attended with any benefit.

This point will be further considered in the short notice that follows the treatment.

Leeching the epigastrium was in all these cases, with but two exceptions, in which venesection was instituted, the principal remedy : rarely one application was sufficient, but more commonly it required relays of leeches applied on alternate days for several successive times, before any great impression was made on the disorder, with this, when it could be enforced, a gruel diet was rigidly adhered to. With the exception of this local depletion and of the exclusion of all tonic remedies in this stage, the treatment was similar to that already detailed under the foregoing division ; of which subordinate part of the treatment, moderate purging, more commonly by mercurials than with the former cases, was found most serviceable. In one instance, of a girl, an emetic was of itself found adequate to remove the disorder. But the circumstance of most importance to insist upon, is the necessity of not prolonging this kind of treatment too long; nor did the symptoms alone always indicate clearly when such treatment should terminate: marked relief might have been procured by the leeching and abstinence, but still the sensibility and pain at the epigastrium would persist, and the tongue, even if less red, would still be foul, and the disorder would continue stationary under the continuance of the same plan of treatment, and would only be exchanged for decided convalescence by a timely recourse to a more tonic treatment, and especially the employment of bitter infusions, and by returning at the same time to a more generous diet and perhaps of animal food —but, as, with regard to the medicinal remedies, the choice in gastric affections of those that are appropriate must of necessity often be tentative, the same likewise holds good with the reginimal management of them, and especially as concerns the period for improving the quality of the patient's diet.

In the cases of gastric affections, hitherto recorded in this division of the subject, neither the severity of the actual symptoms in the acute, nor the inroads on the constitution in the chronic cases, were such, as to lead to the inference, that there existed acute inflammation of the mucous membrane in the former, or disorganization of its tissue in the latter. But in the cases to which the remarks immediately ensuing appertain, one or the other of these conditions respectively, in the acute and chronic instances, was either presumed to exist or was verified by necroscopical examination. It is deserving of notice that these proper inflammatory or disorganizing affections, though the cases are not numerous, were with one exception met with in males, while the preceding cases were almost exclusively of females.

*Acute Gastritis.*—Two instances only occurred of acute inflammation of the gastro-mucous membrane, in both, the presence of such inflammation being inferred from the severity of the symptoms, and in one of them confirmed by the necroscopical appearances.

The first case occurred in a man notorious for his pugilistic and intemperate habits ; who, after drinking, but without being intoxicated, was seized with intense pain in the epigastrium and left hypochondrium, momentarily aggravated and attended with tenderness in the same situations, increased by inspiration ; also with bilious vomiting; the tongue was loaded with white fur, but with its surface red beneath, there was considerable fever and constipation of the bowels; prompt application for relief having been made, leeches largely applied to the epigastrium with a soap enema cut short the severity of the attack, which was afterwards speedily terminated by laxative medicines and abstinence.

The second case terminated fatally; from the obscurity of its cause, extreme severity of the symptoms, and singular condition of the whole surface of the body, as well as from the necroscopical appearances, it seemed worthy of being subjoined in more detail.

Case.—*Extreme agony at epigastrium and in loins—purple color of the whole surface of the body during life—excessive redness of the gastro-mucous membrane—redness of the internal lining of the arteries—ecchymosis of the kidney.*

J. H. æt. 30, laborer, was admitted I. P. Oct. 22, shivering, and suffering extreme agony from pain referred to the loins and across the epigastrium. His face and the whole surface of his body, and more especially the hands were uniformly of a dark red color, not very unlike port wine stain. The tongue was contracted and of an ashy whiteness, while the fauces were red without any swelling. The abdomen was tense and tender on pressure. The respiration laborious and interrupted—the pulse 96 and small.

The account he gave of himself was, that he had been suddenly seized on the 19th with great pain, especially in the loins, and that he had vomited since, whatever he had swallowed; he had had no evacuation from the bowels since two hours after his seizure.

He was bled to ℥xxv. without any abatement of his suffering; a warm bath afterwards relieved him slightly. He died very suddenly in the evening of the same day without any symptom of collapse, but having a short time previously expressed himself as being easier.

*Post-mortem examination, Oct.* 23.—The mucous membrane of the stomach was uniformly of an intensely dark red colour; the redness did not extend to the small intestines, nor to the œsophagus. The internal surface of the arteries presented also a dark red color, extending throughout their entire circumference—the blood was diffluent—there was an ecchymosis in one of the kidneys.

M. Lombard of Geneva, who was accidentally present and saw the stomach, considered its appearance to be similar to that met with from acrid poison—no clue to any such origin of the complaint was discovered, and the stomach had been emptied previous to the patient's admission. Dr. Haviland has since met with the same color of the surface of the body in a case of small-pox, which did not terminate fatally. In another instance in the practice of the same gentleman, but where there was no variolous eruption, most of the internal organs of the thorax and abdomen were found to present the same dark red appearance as described in the above case.

*Organic Diseases of the Stomach.*—The next subdivision of cases comprises those (eight in number, seven male and one female) in which the tissues of the stomach were presumed, or, as in one instance, found to be disorganized. The circumstances, on which this distinction from the previous cases was founded, were the following.

1st. The age of the patients, who had all passed the meridian of life (4 of the 8 being æt. from 60 to 62, and 4 from 42 to 48), while their aspect was more aged than their years.

2nd. Family predisposition. This was traced only in one of the patients, æt. 59, whose mother, sister and brother, were stated to have died of the same disease at the several ages of 63, 66, and 69.

3rd. Peculiarity of the complexion; which was most commonly straw-colored, with a haggard countenance.

4th. Gradual approach of the disorder for a length of time, perhaps for several years.

5th. The epigastrium drawn in.

6th. Some circumstance in addition to the ordinary symptoms of gastric disorder, which served to individualize each case; as one of the following. Hæmatemesis, sometimes with melæna, having occurred more or less frequently at some period of the disorder (in 3 of the cases)—pyrosis long continued and copious, (also in 3, including the case in which the disease was verified by inspection)—exact localization of the epigastric pain opposite to the pylorus and reaching to the back—the intensity of the pain and its uniform coincidence with the period of digestion—vomiting more abrupt than in ordinary cases of dyspepsia and independent of nausea, but preceded by paroxysms of severe pain; some constant fixed and uniform morbid sensation in the epigastrium, as of coldness, &c.

7th. The inefficacy of remedies in effecting any change in the character of the gastric symptoms—their power being limited to producing temporary alleviation.

The tongue, in the cases taken collectively presented no uniformity of appearance which might serve to assist the diagnosis; although in each case individually its characters were very constant. Clay-colored evacuations were common in the cases in which the symptoms of organic disease were most marked. Little unfortunately need be said of the treatment. The utmost attention practicable was of course paid to diet; but, however rigorous, this would often fail to procure any alleviation: cold meat in very small quantities was found occasionally to agree best; in other instances no animal food could be borne. In more than one of the cases a pill consisting of equal parts of extract of rhubarb and extract of hyoscyamus was found for a time, if given about an hour after a meal, to render the completion of digestion less painful; and, in several of the cases, *extr. hyoscy.* gr. ij. *and pil. hydr.* gr. ss. having been taken two or three times daily, and continued for a week or ten days, the patients were enabled to be discharged as benefited. In one case, in which hæmatemesis had previously occurred, and the locality of the pain was accurately defined, a single application gave immediate relief to this symptom. As the bowels in all the cases were habitually torpid, the aperient best tolerated was castor-oil. In one instance, as it was believed, of confirmed pyloric disease, a seton in the epigastrium was followed by manifest alleviation of the symptoms, but which did not long endure. Hydrocyanic acid was also occasionally serviceable in allaying the pain.

The case which terminated fatally while under observation is here subjoined.

CASE. *Carcinoma of the Pylorus—Atrophy of the Liver.*—W. B., æt. 60, labourer, was admitted, O. P. Oct. 8, of a pallid but streaky complexion: he complained of constant uneasiness at the epigastrium, which he described as a sensation of something "turning over" in his stomach, with a feeling of weight there: the epigastrium was slightly tender: immediately after a meal, especially of meat, he suffered severe pain in the same situation; milk and rice caused least suffering: he stated also, that, just before passing urine, the uneasiness at the epigastrium was much increased and again subsided on having voided it: frequently on first rising in the morning he vomited a tea-cupful of clear, tasteless watery fluid. The surface of the tongue was generally clean but with two whitish lateral streaks. The bowels were never open without the aid of medicine: the pulse was not accelerated. The complaint had been gradually coming on for two years or more; during the last twelve months only had the symptoms been severe.

From the 8th to the 29th the treatment consisted chiefly in the application of tartar-emetic ointment to the epigastrium, and in giving gr. $\frac{1}{8}$ doses of acetate of morphia and occasionally ol. ric.—during this period his sufferings had somewhat abated, and the pyrosis but rarely returned. In addition to the above symptoms, he now complained of incessant calls to pass his urine, which continued afterwards to be a very frequent symptom. From the 29th of October to the 19th of November the same plan was continued, with increased doses of the morphia, and a substitution of the decoct. aloes comp. for the ol. ric. During this interval his sufferings had again become more severe, and the pyrosis returned frequently; and during the last week of this period he had no evacuation from the bowels, and every thing he swallowed caused vomiting; the pulse had now become much accelerated and quite thready. He was then urged to become I. P. but declined, and remained at home without further attendance at the hospital till his death, which took place on the 13th of Dec. His friends maintained that, during the last five weeks of his life, he took nothing but cold water, or a little tea.

*Post-mortem examination, Dec.* 14.—Thoracic organs generally healthy—muscular parietes of heart rather soft. Lungs remarkably pale, their vesicular structure very perceptible from the size of the cells.

The stomach contained a pint of dark fluid—the cardiac extremity, and the whole extent of the mucous membrane, to within about four inches of the pylorus, appeared healthy; the membrane admitting of being torn off in moderately large shreds. The pyloric extremity presented one entire ulcerated surface, dark, uneven, with abruptly defined edge, resembling much the dysenteric ulcer of the rectum—the little finger could not be passed through the pylorus, without rupturing it—all the remaining coats of this portion of the stomach were excessively thickened, and in some places almost cartilaginous; in others gelatiniform and easily lacerable: indurated, cartilaginous, tuberculated glands surrounded the diseased portion of the stomach, so that the whole formed a large dense mass. There was no other disease in the intestinal canal. The liver was globular in form, and so contracted as to be entirely confined to the right hypochondrium; it was very hard, but otherwise its section presented nothing remarkable.

*Hæmatemesis.*—Although hæmorrhage from the stomach has but rarely occurred, during the period of these observations, in any case to such an extent as to appear to constitute an affection *sui generis*—yet, as an incident or symptom, in the course of several of the cases, it has not been so infrequent as not to merit a brief notice. Indeed, in several cases, the presence of this symptom has been the only tangible circumstance by which to connect their histories, and introduce them in this clinical analysis. Viewing it, therefore, chiefly as incidental to other affections, it may be most convenient to state, generally, the different descriptions of cases in which it occurred. The total number of cases in which it was noticed did not exceed 11.

1st. In cases of merely functional disorder of the stomach.

In two out of the 101 cases, the subject of analysis, in the early part of these pages, and which were characterized by irritability, as well as morbid sensibility of the stomach, *i. e.* in cases of dyspepsia with vomiting, hæmatemesis and melæna had occurred in the previous history of the patients. It also formed the principal feature in the case of a married woman, æt. 54, in which, likewise, symptoms of functional disorder of the stomach, with nausea, but without vomiting, except of the blood, were present; with costiveness of the bowels. The complexion was wan and sallow, the circulation normal; but there was a dry cough and dyspnœa. There was considerable difficulty in determining the source of the hæmorrhage; the patient recovered under the use of small doses of calomel and colocynth.

2dly. In cases terminating in disorganization of the tissues of the stomach.

In three out of the eight cases, lately reviewed, in which this condition of the stomach was inferred, hæmatemesis with or without melæna (pitchy stools), had occurred in the course of the disorder.

3rdly. Cases of amenorrhœa, in which the gastric hæmorrhage seemed to be connected with the suspension of the catamenial discharge. Three cases of this description occurred. (1.) One in an unmarried female, æt. 21, in service, of a robust habit, yet with a pale and sallow countenance—subject to ordinary symptoms of stomach disorder, but more especially to tympanitic swelling of the abdomen; with a very excitable circulation—there were cough, dyspnœa, and formerly slight hæmoptysis, auscultation furnishing only negative results. Amenorrhœa had existed for some months, and latterly there was a thick vaginal discharge, with much redness and tumefaction of the nymphæ, and termination of the urethra. In the course of the disorder an erythematous eruption of the thighs appeared. In this patient slight hæmatemesis recurred at intervals, and, according to the patient's account, at the period when menstruation was due. But little benefit was derived from any remedies: the patient married shortly, when she became affected with chronic eczema of the scalp, which still continues. (2.) The second was of an unmarried female, æt. 24, in service, possessing embonpoint, but of a delicate appearance; subject to a dry cough with dyspnœa, and of a phthisical aspect, but nothing being detected by auscultation beyond an occasional rhoncus: during treatment in the hospital, she had hectic fever, with stomach disorder and confined bowels: she had not menstruated for three months. In the course of her complaint, she several times vomited blood in considerable quantities of a florid color—she recovered by mild purging followed by quinine—the pulmonary symptoms had early disappeared. (3.) The third case was of a widow, æt. 27. In this patient irregular ague, of a month's duration, had preceded the hæmorrhagic disorder, but had left no sensible enlargement of the spleen: during the course of the latter affection, the pulse continued very variable in force and frequency, with palpitation and œdema of the legs, of one, more especially: there were likewise cough and dyspnœa, and frequently slight hæmoptysis, but still the prominent symptoms were those of disturbance of the gastric functions, with hæmorrhage from the stomach: the bowels were generally torpid: the catamenia had been interrupted for six months. In this case, the hæmatemesis was frequent, and followed by "sooty stools" (melæna). The remedies employed were purging with calomel, saline and vegetable diuretics, with tinct. lyttæ. Under this treatment she recovered, and during her recovery the catamenia were fully re-established.

4thly. In cases of visceral disease of the abdomen, most probably of the spleen—one instance only of this description occurred; but, considering that its history remains incomplete, and that it was too complicated to admit of a connected abridgment, the heading of this eventful case, as entered in the case-book, may suffice to convey some idea of the extent to which the hæmorrhage was carried, and the probable kind of disease with which it was associated.

*Case.* A married woman, æt. 33,—ague for 12 months—an undefined tumor, occupying the left side of the abdomen, producing an induration principally in the left iliac region—abdominal disease had been progressing for four years, dating from the ague—an intercurrent uterine affection, treated, according to the patient's account, by "removal of something from the womb," in a London hospital—latterly hæmatemesis, extremely copious and frequent—almost daily for many weeks—with occasional melæna and hæmaturia—much suffering from pain and tenderness in the indurated region of the abdomen, and in the epigastrium. The hæmatemesis was successfully combated by small doses of calomel and opium, acetate of lead, and sulphate of copper, given in succession—extremely severe and copious salivation from mercurial inunction, continued only five days—only temporary interruption of the hæmatemesis—the ptyalism relieved apparently by lead and opium—subsequent treatment of the general disease by iodine—recurrence of the salivation without a repetition of the use of mercury—occurrence of severe diarrhœa, with final cessation of the hæmatemesis—further improvement to the local tenderness by a seton—continuance of the iodine treatment. At the time this patient, who was made O. P., discontinued attendance, the disease appeared to be checked and indolent.

5thly. *Idiopathic (?) Hæmatemesis.*—One case remains to be mentioned, in which the gastric hæmorrhage could not be attached to any concurrent affection, and seemed, therefore, an idiopathic disorder—the case on this account is subjoined—attention is invited to the circumstance, that the internal hæmorrhage was so sudden and copious as to produce syncope, and to be followed by the usual symptoms ensuing upon excessive loss of blood.

CASE. *Hæmatemesis and Melæna.*—G. B. a labourer, æt. 42, was admitted

I. P. July 30. Spare, and of a slightly yellowish complexion. Tongue was loaded with white fur—the appetite moderate, epigastrium rather sensible to pressure, and bowels rather confined; pulse normal; his chief complaint was of a "heavy beating" at the pit of the stomach with soreness there and at the chest. The following was the account he gave of his disorder: he had been ailing two months with a nipping pain in the abdomen, and about the 10th instant suddenly fainted, and shortly after had purging, the stools being as black as pitch, which continued for two days: ever since the stools had been very dark. On the 16th he vomited more than a pint of blood, and on the same day, had again pitchy stools—since that time he had felt very weak. During the first three days succeeding his admission, he took each night calom. gr. v., and occasionally had ol. ric. and was put on a milk diet. During this time the stools, at first very dark but not loose, became looser and much less dark—he had all along complained much of pain in the forehead and vertigo—he was then ordered calom. gr. ij. o. n. and ol. ric. p. r. n. and was allowed meat diet—by the 29th he was much improved in his health: the stools were relaxed and of a bright yellow colour—the same treatment was continued with diminished doses and gradually left off, and he was discharged well on the 8th of the following month.

### Disorders of the Intestines.

The same division of cases has been adopted in regard to intestinal affections, as was chosen in those of the stomach, viz. into non-inflammatory or functional, and inflammatory or disorganizing, and with the same reservation as to the grounds of diagnosis.

### *First Division of Cases, in which neither Vascular Congestion nor Inflammation were supposed to have existed.*

*Constipation.*—Constipation as the simplest form of functional derangement of the intestines, may first be noticed.

*General Statement.*—Of the total of cases entered in the hospital registers in the years 1836-37, 2.18 per cent. are included under this head; and in 1837 (of which year the data are more complete) not 1 per cent. of the male cases were of this description, while this complaint constituted 3½ per cent. of the females. As to their proportional distribution between the town and country admissions of 1837, 2.75 per cent. of the former, and 1.35 per cent. of the latter, were cases of constipation.

*Analysis of Cases.*—For so simple a disorder as constipation it is not to be expected that there are many applicants to the hospital, till, by its continuance, it has become complicated with some ulterior disorder, producing pain or much inconvenience: up to a certain point, its treatment remains in the hands of the patients' themselves or of druggist-practitioners; and it is, not improbably, to this circumstance that its subsequent aggravation is in many instances attributable—and hence, in hospital practice, it is the result of the treatment rather that suggests the diagnosis of so simple a form of disorder, being the origin of the evil, than the actual symptoms in its complicated state.

These remarks may suffice to shew, that, in attempting to extract from the cases (36) of this affection in the charge of the author anything deserving of notice, in regard to its predisposing causes, symptoms, or treatment; the denomination of constipation, as the substantive affection, was in many of the instances adopted on somewhat arbitrary grounds; and especially of that subdivision of them, which may be qualified by the title of *constipation with dyspepsia.*

As disorders of the stomach and of the bowels are naturally allied in their functional relations so are they in their predisposing causes. Agreeably to this obvious proposition, the circumstances attending the origin of the disorder in the present cases were similar with those enlarged upon in the preceding analysis of cases of functional disorder of the stomach. Residence in a town (two thirds of the cases being of town patients); the habits and confinement within doors of domestic female servants; excessive sedentariness and want of fresh air on the part of those living on the use of the needle; abuse of purgative medicines; the epochs of puberty and of the climacteric age; parturition, lactation, precursory ague, or aguish diathesis; convalescence from continued fever—such were the various circumstances to which the origin of the disorder appeared on investigation to be attributable. It would be useless attempting to explain why, with an apparent identity in the predisposing causes, the resulting form and seat of the affection should not have been identical likewise: why the derangement should have fixed upon the first step in the process of assimilation in the one class of patients and on the later stages in the other. But, setting this question aside, in viewing the history of the cases, a difference may be traced in the ulterior results corresponding to this difference in the preliminary disorder: for, if in both description of cases, in the gastric and in the intestinal, the more remote parts of the economy resented the derangement of the primæ viæ, it was not to the same extent in both; rarely was there that extreme disorder of the circulating system noticed in the present instances, which was so common in the gastric cases; in a single instance only had it arrived at the degree of producing anæmia. The patients for the most part retained their embonpoint; although with few exceptions paleness or discoloration of the countenance characterized the disorder in its most usual chronic form. Loss of strength moreover, although not uncommonly, was not so frequently complained of, as by those whose cases have been referred to the subject of gastric disorder. Thus far then, the degree in which the general health had suffered, as conveyed in this cursory statement, is consistent with the diagnosis formed of them, which implied that the primary stages of assimilation, viz. chymification and chylification, having been duly performed, the ulterior derangement in the digestive apparatus, in the intestinal canal, was attended with less prejudice to the animal economy. Some remarks on certain peculiarities in the immediate and remote symptoms or disorders of functions, to be made in the further analysis of the cases, will be found in accordance with this view.

If all the (36) cases have been included under the head of constipation, as the substantive disorder in each, still they might have been subdivided, according to certain varieties in the proper intestinal symptoms, or in the nature of the secondary or dependant functional disorders.

And first of the symptoms, proper to the intestines themselves.

*The Constipation.*—Every grade in this derangement of the intestinal functions was met with; as simple torpor of the bowels requiring the continued solicitations of medicine—daily but scanty and indurated evacuations—massy and copious fæces voided at protracted intervals—obstinate obstruction of the bowels, in several instances lasting for a week, and, in one case, an entire fortnight. But one remark need here be made on this part of the subject, viz. that although the longer a constipated habit of body had lasted, the greater was the mischief that ensued to the general health, still the cases in which the fæces had accumulated in the canal to the greatest amount or for the longest space of time, were not generally those in which the health was most deteriorated—a circumstance to be explained perhaps, by admitting that in such cases while defecation was simply at fault, the proper nutrition of the body had been ensured by all the previous steps in the process of assimilation.

*Pain and Tenderness.*—Sometimes there was increased sensibility to pressure all over the abdomen, especially in children. In one instance this existed to such a degree that leeches were prescribed, yet entirely disappeared on free evacuation of the bowels before the application of the leeches. The pain with or without tenderness usually corresponded to some portion of the colon, more particularly the ascending or descending portion. In other instances both hypochondria were painful, and excluding the cases in which gastric disorder prevailed, the right hypochondrium more frequently than the left. The umbilical region was rarely painful, and about as seldom was there pain and increased sensibility of the hypogastrium. But perhaps the most frequent seat of pain was the loins, extending forwards to the front of the abdomen ; that this resulted from the state of the ascending or descending colon was clear from the relief consequent on the simple evacuation of the bowel. (It may be here observed that the loins are the seat of pain in as great a variety of disorders as almost any part of the body : the pain apparently depending in different cases upon the condition of very different organs, although vaguely referred alike to the lumbar regions, or by the patients themselves to the kidneys ; as, *e. g.* in rheumatism, renal disease, disorder of the menstruation, especially menorrhagia, the gravid uterus, piles, constipation : as a point in diagnosis it is by no means immaterial to determine its source). The interscapular region was not an uncommon seat of pain also, in these cases of constipation. Another description of pain, which, although not corresponding to the region of the colon, seemed to result from its distention by fæces or flatus, was cramp in the legs; in two instances this was the prominent symptom and yielded to the free evacuation of the bowel.

*Flatulence.*—General tension of the abdomen, local tympany over the colon and especially the caput coli, borborygmi, large extrication of flatus with the stools, fulness of the hypogastrium, were the various modes in which flatulence of the intestines occurred. Over-distention of the canal by the gaseous products of the intestines seemed in many of the instances the cause of the constipation.

*Disordered Secretions*.—As far as opportunities of ascertaining this point existed, the change in the appearance of the fæces was more commonly the result of protracted absorption of their more fluid part than from altered secretions ; where these were much altered, the liver seemed to be implicated, as where the stools had a clayey appearance. In one instance a sudden and excessive secretion of bile into the canal seemed to act as a spontaneous purge to dislodge the accumulated fæces.

*Symptoms or Disorders referrible to other Organs or Functions. 1st. The Stomach.*—Unquestionably many of the present cases, if their nature had been estimated merely by the more obvious symptoms and the complaints made by the patients, would have been included with those reviewed as instances of functional disorder of the stomach. The following were the grounds on which the gastric symptoms were assumed to be secondary to the intestinal, in the cases at present under review. Although the gastric disturbance, when present to any extent, coincided in its general phenomena with the cases of primary affection of the stomach, yet it was distinguished by its being terminated by the free evacuation of the bowels : some of its symptoms, however, in their individual bearing likewise presented some points of distinction. Vomiting for instance was more frequent than prolonged nausea; and occasionally seemed to be the result of inverted peristaltic action consequent on the ineffectual efforts to dislodge the fecal contents, and in one instance the concussion produced by the violence of the vomiting appeared to have this desired effect. The uneasiness attending digestion was situated in regions corresponding to the colon rather than to the stomach—the pain in the left hypochondrium, the "side-ache" so often complained of, and so rebellious in cases of dyspepsia, yielded in the present cases readily, when the bowels acted freely. As some degree of constipation is as common an accompaniment of dyspepsia as dyspepsia is of constipation, it might at first sight be said that the point of diagnosis under consideration is immaterial to the treatment—this is however far from the truth; for the most mischievous practice in dyspepsia accompanied by constipation is indiscriminate purging, whereas in the present disorder much greater latitude is allowable in the use of purgative medicines, but of this hereafter.

*Circulation.*—Occasionally an extremely rapid pulse, frequently more or less palpitation, syncope in one case of a delicate female during lactation, and in another patient constantly employed at the needle, anæmia, were among the secondary affections—but that extreme disturbance in the circulation so common in the cases of stomach affections was rarely met with in these; neither were febrile accessions so frequent; œdema was not uncommon, confined chiefly to the lower extremities, and sometimes to one leg: this seemed to be of a different nature from the puffiness of the ancles, met with so frequently in asthenic females; as it occurred as often among the robust, and was rather of the nature of that which accompanies erythema nodosum, into which in some cases it seemed to pass, and it was generally coincident with amenorrhœa.

*Pulmonary System.*—Cough and dyspnœa occurred but rarely in comparison with the gastric cases.

*Nervous System.*—The most frequent were the affections of this system and which were characteristic of a certain portion of the cases—vertigo, head-ache, and drowsiness, being the ordinary form—hemicrania, wakefulness; heaviness of the limbs; aching of the legs; tremors; in one instance, of a child, an approach to chorea; in another hysterical fits; in several depression of spirits, more common perhaps at the climacteric epoch, were likewise observed. Dimness of sight was complained of by some; in one instance of a young girl constantly employed at her needle, this, accompanied by a troublesome dazzling, was her principal complaint, but yielded with the intestinal disorder.

*Menstruation.*—The catamenia were not deficient in the same proportion of cases as in those of gastric affection: occasionally they were even profuse: when not absolutely wanting, this defect consisted rather in the irregularity of their returns than in scantiness, or discolouration—some of the cases, however, seemed to be characterised by the presence of amenorrhœa as a complication; in these the cessation of the catamenia was often sudden and not preceded by gradual failure; and their return was often as abrupt at an early period of convalescence; a circumstunce in contrast with the cases of prolonged gastric disorder in which the re-establishment of the catamenia was postponed to a much later period. A pricking sensation of the mammæ was felt in one instance in connexion with the suspension of the catamenia. In more than one instance among the unmarried females applying for relief for constipation accompanied with dyspeptic symptoms, including vomiting and amenorrhœa, a gravid uterus was the probable cause of the disorder.

*Treatment.*—Much will not be expected to be said on the treatment of so simple a pathological state as constipation—yet, as in the majority of the cases the patients of their own accord or by the advice of others, had for a considerable time taken purgative medicines previously to their application at the hospital (the common excuse for such application being that they could not afford to pay for more medicine) and their treatment as hospital patients still continuing to be essentially purgative, it may be worth while to enquire to what modification of this common treatment, it was owing, that a more prompt and effectual result was in the latter instance ensured. " Salts and senna" are the ordinary drugs employed by the poor in their own houses, and often in no sparing measure, whereas these remedies were but rarely employed in the present cases after their becoming hospital patients ; but on a general review of the treatment adopted, its principal peculiarity and contrast from the domestic management, consisted, as the notes of the cases declare, in the frequent combination of tonic remedies with those of a purgative nature. Of these tonic remedies the principal and almost sole, was sulphate of iron, in combination with aloes or extr. coloc. comp. The purgatives employed were chiefly of the resinous class—and as a general remark it may be noted, that the more chronic the primary disorder of the bowels or the more advanced and grave the disturbance of other functions, the less could simple purgatives be depended on, and the greater the necessity of employing tonics and the larger their share in the success of the treatment. The one of iron was by no means beneficial only when it might be expected to act as an emmenagogue ; unusual torpor of the colon, or debility of digestion seemed equally to require and be benefited by its combination with purgatives. The history of the cases further evinced the good effects of varying the drug in different cases or in the same case at different stages ; to detail these varieties of treatment would be to little purpose. The above brief exposition, pretends only to express the general character of the treatment, with a view of recommending a seasonable combination of tonics with purgatives in such class of cases : neither is any claim to novelty preferred in this respect ; but the object has been merely to add the results of a trial on a tolerably extensive scale,* to those with which the profession may be already acquainted in favor of this modification of treatment. Leeching in the vicinity of the external labia sometimes aided the return of the catamenia. Blisters to the nape of the neck or behind the ears generally relieved the cerebral symptoms. Leeches or other depleting remedies were rarely required for the removal of any local pain.

*Intestinal Entozoa—General Statement.*—The number of cases, entered under this title in the hospital registers during the years 1836-37, amounting only to 0·69 per cent. does not include all in which these entozoa were known even to have occurred ; since their presence in the intestinal canal was, more frequently than not, merely an adventitious circumstance in the course of graver disorders ; also many cases have been registered under other titles, as chorea and epilepsy, although these affections may have been caused by the presence of worms.

*Analysis of Cases.*—The number of cases of which the notes have been kept during this period amounts to 17—8 being cases of ascarides, 6 of ascaris lumbricoides, 2 of ascar. and ascar. lumbric. co-existing in the same individual, and 1 of trichocephali—12 were of females, and 5 of males. If an estimate made on so small a scale might be trusted, it would seem 3 cases occurred among country patients to 2 among town patients ; and taking the proportion of the totals of male and female admissions, that rather more than three females were subject to these entozoa to 2 males. With respect to age, 3 of the patients were 10 years or under, 7 from 15 to 20, 5 from 20 to 40, and 2 from 55 to 65.

*States of Health or Diseases in which they occurred.*—Three were cases of functional disorder of the stomach, one of gastric disorder with inflammatory

* The number of cases from which the results of this generalization have in strictness been deduced, was only 36 ; but it may be added that they are in exact unison with the results of a much more extensive adoption of the same treatment, both previously and subsequently—but of which no precise notes have been preserved.

symptoms; 2 of severe inflammatory disorder of the intestines, one of which terminated fatally with disease of the liver; one, the case of tricocephali, occurred in a child æt. 4, who died of fever, and in whom several of these entozoa were found lodged in the cæcum and appendix vermiformis. As in none of these 7 cases did the principal affections appear to be either maintained by the presence nor terminated by the removal of the entozoa, no further notice of them need here be introduced. Of the remaining 10, one was attended with epilepsy, 3 with chorea, 1 with tremors of the muscles, 1 with symptoms of cerebral congestion (?) and 4 with various symptoms, direct or sympathetic, but of no great severity. As in each of these latter 10 cases the concurrent affection seemed to depend more or less upon the presence, at some period, of the entozoa, and in most of them readily subsided on their removal, they may with propriety be here reported as proper cases of parasitical affections.

*Accompanied by Chorea*—all female patients. In 3 out of 6 cases of chorea, worms) ascar. lumbric.) had been passed from the intestines. Two out of the 3, patients had just reached the age of puberty; menstruation in one occurring for the first time during convalescence from the disorder and while taking chalybeate medicines. In the other, the menstruation was normal, but only recently established. The third, aged 13, had never menstruated. In none of the three was there much general disorder of the health nor even of the digestive functions, slight emaciation with discoloration of the countenance being principally remarked. The chorea affected both sides, and was of considerable virulence in each case. In none of them did purgatives with oil of turpentine alone succeed in controlling the chorea, although they might have dislodged the worms; but in two out of the three cases the chorea gradually disappeared in the course of three weeks or a month under the use of chalybeates with the shower-bath: but neither, on the other hand, did these last-mentioned remedies check the chorea till the worms had been removed. In the third case of the child under the age of puberty, chalybeates had no effect, but recovery ensued on the use of the shower-bath with an occasional aperient. From these three cases then it would appear that, if the presence of ascar. lumbricoid. in the intestinal canal be adequate to produce the irregular movements of chorea in the first instance, the convulsive affection may be subsequently maintained without their presence, it would appear probable that some such predisposing condition as the epoch of puberty, and family tendency (the sister of one had been similarly affected) is necessary, as a concurrent cause in the production of chorea.

*Accompanied with Tremors.*—The history of the case thus circumstanced, was briefly the following. A girl, æt. 11, had for more than two years been in the habit of voiding ascar. lumbric. at various intervals. A short time after their first appearance she fell out of a cart, but made no complaint at the time; in a few days she became affected with tremors of the upper extremities, such as continued at the time of her admission at the hospital; they had left her for periods of a month or more since their first occurrence; these tremors ensued only on her holding or grasping any thing in her hand; there were none of the irregular movements of chorea; her health did not otherwise appear to suffer. During the fortnight she remained under treatment, which consisted in giving ol. ric., ol. terebinth, and ferri subcarb, worms were occasionally voided, and the tremors had disappeared—the case was subsequently lost sight of.

*With Epilepsy.*—A robust young man, æt. 21, had been subject to epileptic fits for rather more than twelve months, sometimes daily, sometimes at intervals of five or six weeks; three or four months previous to his admission he had passed several worms of the species ascar. lumbric., and only three days previously had voided an immense number at a single evacuation, according to his own statement as many as 100. He had had no fit since this occurrence, neither was there any return of epilepsy during his continuance for some time in the hospital on account of a different malady. The fits were generally preceded by an attack of rigors, and followed by headache.

*With Symptoms of Cerebral Congestion.*—A labourer, æt. 35, complained of pain in the left temple, noise in the left ear, vertigo, and habitual drowsiness; his bowels were alternately loose and bound; his meals unsatisfying—he was cupped at the nape of the neck, blistered between the shoulders, and had a calomel and jalap purge, which relieved the cerebral symptoms; during twelve days he continued to take slightly aperient medicine, when it transpired that he had lately passed large numbers of ascarides: the same treatment was continued with the addition of bitter enemata: in another week he was free from complaint. One of the remaining four cases, a case of ascarides, was somewhat similar to the foregoing. The patient was a man æt. 60; he suffered intense headache, and had considerable gastric disturbance. Sulphur electuary and bitter enemata removed the ascarides, but the other symptoms did not immediately subside, but yielded subsequently under the use of the decoct. aloes comp.

The remaining three cases need be but briefly noticed—two of ascarides—one occurred in a delicate woman æt. 24, during lactation, with a tendency to syncope and some gastric disorder, she recovered on weaning her infant, and taking pil. hyd. and aloes in small quantities with the use of bitter enemata—the other was of a child æt. 2, with constipated bowels; the complaint yielding to the use of hyd. c̄ cret. and ol. ric. The remaining case was of asc. lumbric. occurring in a feeble scrofulous child of an unhealthy mother, and was attended with some headache, disturbed sleep, an unnatural appetite, and constipated bowels; the child recovered under the use of gentle purging with a simple application of leeches to the head.

*Diarrhœa.*—Diarrhœa may serve as the connecting link in this arrangement, between those disorders of the intestines, in which there was no reason to apprehend the circulation of the blood in them to be augmented, nor even there to have been any congestion of the bloodvessels; and those affections, in which one or other of these conditions might fairly be presumed to be present. For cases of diarrhœa might probably belong to one or other of these classes according to circumstances, although it is not easy to decide always from the symptoms, to which category each individual case may belong.

*General Statement.*—Excluding those cases in which diarrhœa was present merely as the common attendant of the continued fever of this country; and also those cases, in which it occurred, only as the accompaniment of the advanced stages of phthisis; this disorder was not very frequent, compared with other intestinal affections; not amounting to more than 1.09 per cent. on the admissions of the two years, (not more than half as common as the cases of constipation.) In 1817 it constituted 0.61 per cent. of the male, and 1.54 per cent. of the female admissions, and was nearly equally distributed between the town and country patients.

*Analysis of Cases.*—The cases falling to the charge of the author during this period not exceeding 27, scarcely admit of more than a brief notice. Few as they were however, they may serve to illustrate the great variety of conditions under which diarrhœa arises: since in only 12 of the 27 cases could it be regarded as an idiopathic primitive disorder of the bowels themselves, originating independently of any predisposing morbid conditions of other organs or of general constitutional disorder. In the remaining 15 cases the diarrhœa might, as appeared from the history of the cases, be attached to one or other of the following conditions—a cachectic state of the tissues of the body—phthisis in its early stage—the aguish diathesis, (in which the diarrhœa alternated with attacks of ague,) parturition, weaning, dentition; derangement of the liver; uterine disorder—erythema nodosum. With regard to the nature of the discharge from the bowels, the usual varieties occurred, without any apparent relation to the nature of the predisposing cause, except where the liver was evidently implicated, in which case the stools were either yeasty with an excess, or pale with a deficiency of bile. The presence of blood in the evacuations was not infrequent, as it was noted in 5 of the 27 cases. Cases in which there was a fixed tenderness referrible to some portion of the intestinal tract, are not here included, as belonging more properly to pure enteritis, in which disease diarrhœa, if present, for the most part alternated with constipation.

In almost all the cases the functions of the stomach were materially impaired—nearly in one-half vomiting occurred at some period of the disorder: redness of the tongue was observed in but three instances. Thirst was more frequent, and it may be noted that this symptom was by no means in proportion to the degree of pyrexia present; indeed it was remarked occasionally to be more urgent where there was least disturbance of the general circulation, and it appeared rather to be the result, as in cholera, of the excessive drain of fluid from the canal. In one case it was much complained of, at the time the tongue continued remarkably moist.

Extreme frequency or excitability of the pulse was the only noticeable derangement of the circulation in about one-third of the cases; in about the same number the disorder commenced by a smart ephemeral fever, or slighter febrile accessions supervened repeatedly during its course, or more or less hectic was uniformly present. In the remainder there was scarcely an appreciable disturbance of the general circulation. These various states of the circulation seemed to occur indifferently under any of the varieties of the discharge from the bowels; with the exception that in all the cases, but one, of sanguinolent diarrhœa, the general circulation was more or less disturbed. The derangements of other systems, of the nervous especially, were not so frequent or so prominent, although of the same character, as in the cases of gastric affections.

*Treatment.*—Notwithstanding this diversity in the predisposing and exciting causes, and in the character of the diarrhœa itself, the treatment by which 24 out of the 27 cases (the remaining three not being followed to their termination) were, both the acute and the chronic, conducted in no long time to a favorable issue, was more uniform than might have been anticipated. Opium in small doses in the form of Dover's powder, for the most part combined with the same quantity of hydr. c̄ cret., repeated more or less frequently according to the number of the stools, was the remedy in which most reliance was placed. The presence or absence of fever did not seem to affect its usefulness: neither did the nature of the alvine secretions demand more attention in this respect. The liquor calcis was found a valuable auxiliary to this the principal remedy. Astringents and the common chalk mixture were rarely employed, though occasionally with benefit. When recent, which few of the cases were, the diarrhœa was sometimes effectually checked by rhub. and the pulv. cret. comp. c̄ op.

*Enteritis and Colitis.*—The next subdivision of cases to be noticed, was contra-distinguished from the foregoing by the occurrence of fixed pain with more or less tenderness over some limited region of the abdominal cavity; and by further evidence of the vascular system of the intestinal canal being interested, as furnished by the good effects resulting from general or local bloodletting. To these the title of enteritis or colitis appeared appropriate, in which some portion of the canal was regarded as in a state of inflammation, including the corresponding portion probably of the peritoneum. The cases in the general practice of the hospital were too few for a statistical account to be given of them—the present remarks relate to six only, under the charge of the author during this period. Exclusive of the fixed pain and tenderness, the symptoms observed resembled those already noticed in the cases of other intestinal affections. In two only could any exciting cause be assigned; one appearing to originate from difficult parturition; the other from drinking cold water during a state of fatigue and free perspiration. Diarrhœa either did not exist or alternated with constipation: the stomach was usually disordered, much as in the preceding cases; vomiting being frequent. Redness of the tongue, when present, occupied the centre rather than the margins, which is in contrast with the cases of gastric affection. In one case the attendant fever much resembled ordinary continued fever; the principal circumstance, distinguishing it from the latter, being the predominance of the local symptoms, with which the patient himself was pre-occupied. Local depletion by leeches, and in the case just alluded to, venæsection, were the remedies chiefly employed, with gentle means for procuring free action of the bowels in the cases attended with constipation, and alternate doses of mercury and ipecacuanha in those accompanied with diarrhœa. In one chronic case, in which powerful antiphlogistic remedies had been previously used, the cure was completed by a seton inserted over the seat of tenderness, combined with occasional aperients.

*Hæmorrhoids.*—Before concluding the section of cases in which the functions of the mucous membrane of the intestines were chiefly interested, accompanied by local vascular derangement; some mention should be made of cases of hæmorrhoidal affections. But few patients comparatively apply for relief for this affection alone; two cases only occurred during this period in the practice of the author at the hospital; one in a female approaching the climacteric age and after prolonged lactation, and was accompanied by diarrhœa; the disease in this instance yielded to gall ointment and the subsequent use of chalybeates—the other was the case of a man aged 40, and was attended by moderate constipation, during the treatment for which, salivation having accidently occurred, he recovered from the hæmorrhoidal affection.

Of the cases hitherto reviewed of disorders of the stomach or bowels, amounting to more than 200, the notes make mention of hæmorrhoids in six only, all females: from which it may at least be inferred that they were rarely present as a prominent or troublesome affection. The ages of these six varied from 35 to 65—with the exception of one, all had borne children; in all the bowels had been more or less constipated; in one the liver was much disordered; another had been long subject to worms (asc. lumbric.); the affection in another already mentioned came on during prolonged lactation.

*Scirrhus of the Rectum.*—A single case of annular scirrhus and stricture of the rectum may be just alluded to (although not conducted to its final issue) on account of nitric acid, and afterwards small doses of sulphate of copper having been found to improve the alvine secretions, which for a considerable time consisted of shreddy sanguinolent mucus of a chocolate color, aggravating in a great degree the sufferings of the patient.

*Peritonitis.*—This disease, including both its acute and chronic forms, constituted during the years 1836-37, only 0.40 per cent. of the registered cases in the hospital.

The notes of two cases only furnish any matter worthy of notice. One of these occurred in a female æt. 27, and was a case of great severity. The peritoneal attack came on eight days after the patient's getting wet in the feet, and remaining in that state the rest of the day. In the interval she had been sensible of no indisposition: she was suckling an infant four months old at the time; and notwithstanding bloodletting was largely performed, followed by leeching, no diminution took place in the secretion of milk, and she was enabled to continue nursing throughout the attack. The treatment was not begun till the 3d day; copious V.S. leeches, moderate purging with neutral salts, effected her recovery. In another case the peritonitis came on after parturition, the patient having suffered much during pregnancy from local pain in the abdomen. It assumed a chronic character, and appeared to occupy principally the hypogastric region: micturition was performed with great difficulty; violent vomiting was also a prominent symptom, which yielded to a blister to the epigastrium; the peritoneal inflammation had been controlled for a time by mercurial inunction, but afterwards became complicated with gastro-enteritis, rendering the prognosis extremely unfavourable—in this state she left the hospital at her own request.

*Abdominal Tumor.*—A case, incomplete in its history, of moveable tumor in the abdomen, may be noticed for some peculiarities attending it. It was of a woman, æt. 44, previously healthy, and who had borne nine children. The tumor first began three years previous to her admission, following her last confinement. At the time of her admission, there was a large and very indurated, but painless tumor, occupying the epigastrium. Subsequently it became very tender, when a severe attack of intermittent fever, of a typhoid type, supervened. The patient would not continue in the hospital. The intermittent was not in the least under the control of quinine.

### DISEASES OF LIVER.

The entries in the registers of cases of disease of liver, inclusive of jaundice, amount to about 1 per cent on the total admissions during the years 1836-37.

The cases, of which the notes have been preserved, are 14; and may be divided, 1st, into those in which jaundice was the principal feature, (4). 2ndly, those in which sanguineous congestion or inflammation of the liver seemed the predominant pathological condition, (5); and, 3rdly, those cases of organic disease of the liver as inferred from the symptoms during life, or ascertained by necroscopical inspection, (5).

*Icterus.*—The 4 cases of jaundice may serve to show, from the diversity of circumstances under which it originated, its indeterminateness as a basis for a nosological distinction.

In case 1, the patient, an unmarried female, æt. 22, had been subject to attacks of jaundice every Spring and Autumn for four years; during each attack the catamenia were suspended; the alvine excretions were natural, neither was there any abdominal pain or local tenderness; the last attack yielded promptly to purging with *pil. hydrarg. and aloes*, and the use of *decoct. tarax.* with *supert.* of *potash*.

In case 2, of a female æt. 50, the jaundice appeared to be evidently produced by obstruction to the passage of bile into the intestines, through the biliary ducts, as declared by the character of the stools, and other symptoms. The disorder had lasted two years, and had been attended with repeated attacks of retching and occasional vomiting of colorless fluid. These attacks, accompanied by pain "drawing her double," were followed each time by a deeper hue of the jaundice. Latterly a spontaneous and more violent attack of vomiting, in which bile was rejected, was followed by manifest amendment; after which the disorder was entirely removed by emetics, with small doses of calomel, rhubarb and aloes, and mild mercurial inunction.

In case 3, of a blacksmith, æt. 40, with the same deficient passage of bile into the intestines, similar disorder of the stomach, as marked by nausea and retching, and the same chronic character of the affection as in the last case; there was an absence of the spasmodic pain in the epigastric or hypochondriac regions, and, in its place, a tensive pain across both hypochondria, with great resistance in the right hypochondrium and dullness on percussion in the lower region of the thorax on the same side. In this case, therefore, the obstruction was probably in the parenchymatous or secretory structure of the organ, rather than in the excretory ducts, as in the last case. In this instance, the general disorder was much mitigated by *pil. hydr. hyosc. and calumba;* and a further improvement ensued under the trial of hydriodate of potash, but the jaundice was not removed.

The 4th case of jaundice occurred in a shepherd, æt. 21, having malformation of the chest, which projected in front, and in its contour resembled the fœtal thorax. The jaundice came on some time after a rheumatic attack, and was attended with cardiac symptoms, especially a loud bellows sound, with the "frémissement cataire." The right hypochondrium was tense and painful, and there was pain in the right shoulder: there was no deficiency of bile in the alvine excretions. But little effect was produced on the disorder by mercurial remedies, and the history of the case remained incomplete.

*Sanguineous Congestion, or Inflammation of the Liver.*—These were cases (5) which differed from the preceding in the absence of jaundice, while they presented a considerable affinity with them in other circumstances, such as—in the condition of the right hypochondrium which, in both classes of patients, for the most part the seat of some morbid sensation, in the present instances was more decidedly tense, painful, and tender—in the occasional occurrence of spasmodic pain in the upper part of the abdomen—in the sympathetic affections of the stomach, viz. nausea and vomiting (in 3 out of the 5 cases). The bowels were more constipated than in the preceding cases, and the constipation more frequently alternated with diarrhœa. The general conditions of the system, under which the disorder originated, were as various as in the cases of icterus. One of the patients was a labourer, æt. 58, who had left India eight years previously, having suffered from "bowel complaints" during the latter part of his residence there, but had since till the last six months enjoyed good health. Tenderness on pressure of the cartilages over the right hypochondrium, nausea, vomiting, spasmodic pain in the epigastrium, constipation alternating with relaxation of the bowels and tormina, formed the principal symptoms, and which yielded to leeches, blisters, and small doses of calomel or pil. hydr. with occasionally ol. ric.—A second case occurred in a married woman æt. 27, of a delicate constitution and while suckling an infant eight months old. The symptoms were such as described in the last case with more disturbance of the circulation characterised by hectic. In this case the urine was loaded with the colouring matter of the bile, although the skin was not discoloured. She recovered under the use of the same remedies with the addition of taraxacum and quassia. Two other cases occurred in robust unmarried females of the respective ages of 20 and 24, in which the disorder was attended with less disturbance of the stomach but more torpor of the bowels, the pain and tenderness in the right hypochondrium being the same. The same remedies, including leeches to the hypochondrium, speedily induced recovery. The fifth and remaining case occurred in a robust girl, æt. 12, in whom all the above symptoms existed in a greater degree, with additional disturbance of the brain, producing head-ache, vertigo and drowsiness. In this case V. S. was employed in addition to the other remedies; the vomiting was controlled by a blister to the epigastrium; the patient recovered in about a fortnight.

*Enlargement or other Organic Disease of the Liver.*—The cases, under this head, were too various in the nature of the organic disease or too complicated with diseases of other organs, to admit of their being viewed synoptically, but individually possessing some points of interest, may be briefly noticed as distinct histories.

CASE 1.—*Enlargement and Induration of the Liver, with deficient secretion of bile into the intestinal canal, terminating in recovery under the use of mercurials and iodine.*

W. B., ætat. 14, labourer, on his admission, April 19, was much emaciated, with a harsh dry skin, slightly tinged, as were the conjunctivæ, with yellow ; the cheeks and lips, and hands, were of a deep purplish hue ; the tongue was white, the appetite gone, and the stools of a light ochry color. The liver was distinctly felt reaching to the level of the umbilicus on the right side, and appeared indurated, with tenderness of its edge. The pulse was 120, thready, but soft.

He had been ill fifteen weeks, being first taken with pain in the right side—the jaundice had been deeper.

Mercurial liniment was ordered to be applied twice daily to the right hypochondrium and a grain of calomel with gr. ¼ of op. given *n. et m.* with *inf. quass. ter quotidie,* and a milk diet. By the 2nd of May, up to which time these remedies were continued, he had much improved, the induration and enlargement of the liver were not easily perceptible ; the urine which had been very high-colored was much paler, the stools contained more bile and the gums were slightly swollen and tender. The same remedies were continued at longer intervals and he was allowed meat on alternate days, but which was found not to agree and was discontinued till a later period. During three weeks there was a further slowly progressive improvement with occasional relapses of pain and tenderness in the right hypochondrium. Hydriodate of potash (gr. j. and afterwards gr. ij. *ter quotidie*) out of camphor mixture was then added to the mercurial remedies, which were still further reduced, and this combined plan was continued till the 17th of June, by which time the liver had become greatly diminished in size, and had nearly retreated to the right hypochondrium, and there was no tenderness although there remained some induration : the stools were natural and the complexion had lost its venous hue. The appetite had long since improved, the stomach tolerated meat, and the boy had become fat—by the 23d of the same month he was discharged apparently well. It may be mentioned that this individual was the brother of the patient whose case was reported in the first part of these *Contributions,* as a solitary instance, among the cases of phthisis, of the physical and other signs of tuberculization of the lung having entirely disappeared in the course of two years.

CASE 2.—*Enlargement of Liver, with jaundice and absence of bile in the alvine secretions—subsequent contraction and induration of the organ with growth of a large cartilaginiform cyst, attended with ascites and general dropsy, terminating in two years from the commencement of the disorder in death.*

The heading of this case comprises the principal points of interest in its history, which was principally remarkable from having afforded the opportunity of noticing the conversion of an enlarged liver into one of normal dimensions but greatly indurated texture. The period of the commencement of the growth of the cyst could not be clearly assigned, nor the question decided whether it preceded or followed the other hepatic disease. The patient was a girl, æt. 12, at the time of her decease. The liver, when she first came under observation (Aug. 17, 1836), reached as far as the umbilicus ; she had then been ill 12 months, and was jaundiced and had very light coloured evacuations from the bowels. Mercurials and iodine did not increase the secretion of bile into the intestinal canal ; but for a time this was effected by a repetition of emetics : no further amendment ensued and the patient left the hospital Sept. 10, and the case was lost sight of by the author till he was invited to attend the post-mortem examination on the 20th of Aug. 1837. She had for many months been labouring under ascites to a vast amount with infiltration of the lower extremities, and extreme emaciation of the upper portion of the body.

*Post-mortem Examination.*—The liver was not larger than natural, and its peritoneal surface pale ; its substance was rigid, and its section mottled with deep red points ; the gall-bladder was full of bile—under its inferior surface and opposite the longitudinal fissure and attached to the liver, was found a cyst of the size of two fists, having firm cartilaginous parietes of the thickness of a half-crown piece, containing perfectly clear and colorless fluid, and which were lined with a soft membraniform white deposit, apparently not organically connected with the cyst, as it readily collapsed into its cavity on the escape of the fluid contents. The internal surface of the cyst, on the removal of the membranous deposit, was rough and of a deep saffron-color, interspersed with spots of a still darker color. The mucous membrane of the intestinal canal presented a glairy mucilaginous deposit on its surface. A thymus gland was found of the size met with in the infant. The heart was very small but healthy, as were the lungs, but the cavity of the thorax was surprisingly contracted.

Case 3.—*Hypertrophy of Liver, with large hydatid cyst imbedded beneath its convex surface. Insidious attack of central pneumonia of the right lung, reaching the suppurative stage, and producing a fistulous opening into the pleura, with pneumothorax, and causing death by inflammation of that membrane.*

C. W., æt. 43, labourer, was admitted I. P. Aug. 11, 1837. He had been ill six months, and during that time had emaciated. He had cough and three or four times had had slight hæmoptysis, but his principal complaint was of pain under the right hypochondrium and extending to the right shoulder. The pulse was small but not accelerated, but he had frequent chills and sweats. The appetite was impaired and digestion difficult, but stools were stated to be natural, while the urine was of the color of porter. The thorax was observed to bulge externally in the inferior part of the right side, where percussion was dull and the respiratory sound absent. A moderate mercurial treatment was adopted without any material effect for a week, when on the 18th he was suddenly seized with rigors and became slightly delirious ; subsequently the breathing became very laborious and distressing, from acute pain in the right hypochondrium, increased by pressure of the cartilages and with much tension and heat of that region. He had likewise vomiting and the pulse had become quick and sharp and hard; by the evening of the following day the space over which the respiratory sound was absent and percussion dull in the right side of the chest had much increased. During this period antiphlogistic means by blood-letting, general and local, had been largely employed, and afterwards acetate of morphia given. The pain and suffering from laborious respiration had then entirely and rather suddenly subsided. The next day (the 20th), the chest was observed to be unusually sonorous where previously it had been quite dull on percussion, the respiration continuing inaudible in the same situation. In this state he continued two days, free from pain or much suffering, but with symptoms of gradual collapse and died on the 22nd.

*Post-mortem Examination 5 hours after Death.*—The bulging on the right side of the chest was very perceptible. On incising the intercostal spaces on that side a large quantity of fetid gas escaped with a slight explosion. On removing the sternum the right lung was found greatly contracted and pushed back against the vertebral column, the surface of the lung was concealed by a thick layer of soft yellowish-white membranous coagulum ; the same description of deposit lined the whole of the left pleura ; the cavity of the pleura was intersected by soft bands of the same material, and contained about a quart of opaque fluid, slightly colored. On exposing the lung and separating the cleft between the upper and middle lobes, a fistulous opening was discovered, from which a large quantity of semi-fluid matter escaped, of a chocolate colour ; this opening led to a large irregular cavity in the center of the lung, in which the pulmonary tissue was softened down to the consistence of pulp : the parts of the lung forming the boundaries of the cavity, though not diffluent, were dark, half-decomposed and easily reducible into pulp by pressure between the fingers—the cavity was intersected by bands of pulmonary tissue in the same state. The rest of the lung appeared condensed by pressure, being tough but pliant. A small insulated mass of cretaceous matter was found in the upper lobe in front—the left lung and the heart were healthy.

On exposing the abdominal viscera the anterior surface of the liver reached below the umbilicus and filled up the epigastric region. Its enlargement had also pushed up the diaphragm into the thorax and caused the bulging—the right lobe was altered in form superiorly, its convexity being much increased (bombée). In this situation was found a large cyst imbedded in the lobe, of the size of two fists and filled with hydatids (acephalocysts), some collapsed and a few entire, and of the size of pigeon's eggs or walnuts. The walls of the cyst were in some portions cartilaginous. The surrounding tissue of the liver seemed healthy ; the gall-bladder was full of a greenish-yellow viscid bile.

CASE 4.—*Vomiting and purging with brief intermissions for thirteen weeks ; stupor the last few days preceding death. Gangrenous and carcinomataus tumor enveloping the gall-bladder, and communicating by two distinct ulcerated apertures with the duodenum and transverse arch of the colon—disease of the mucous membrane of the duodenum and colon—exemption from disease of the jejunum and ilium, but contraction of their calibre (the aliment having probably passed directly from the duodenum into the colon)—copious effusion under the arachnoid.*

M. G. æt. 64, nurse, was admitted Nov. 22, 1837, having had diarrhœa with frequent vomiting for nine weeks. The tongue was dryish and cracked, and the appetite bad. The matter vomited often green and the stools dark—the pulse small and very irregular—features sunken. A defined, roundish, indurated, but painless tumor was detected below and extending beneath the right hypochondrium. From the period of her admission to the 18th of the following month the same symptoms continued with occasional intermissions and but little controlled by the remedies, viz. mild mercurials with small doses of opium, opiate enemata and tonics. The tumor had latterly become very tender. On the 18th drowsiness came on ; the diarrhœa had increased and she appeared sinking, but again rallied during the day, with roaming at night, till the 25th, when she fell into a stupor from which she could be only momentarily roused—this state continued till the 29th when she died.

*Post-mortem examination nine hours after death.* No emaciation.

*Head.* The brain was small and firm ; the arachnoid was raised up in the form of a bladder from the convolutions and was slightly opaque—from 6 to 8 ounces of fluid escaped from beneath it—the convolutions were separated by the interposition of the fluid. The surface was rather exsanguine.

*Abdomen.* Accumulation of fat in the omentum. The liver in its general aspect presented the ordinary nutmeg appearance ; at the lower margin of its right lobe, under the concave surface at the great longitudinal fissure, and extending to the corresponding portion of the transverse arch of colon and the first and second portions of the duodenum, was found a dark, friable, mass of disease, which was ruptured by a slight accidental violence in examining the part, and displayed a fetid, dark gangrenous substance, presenting the appearance of grumous blood being intermixed with the disorganized structure of the liver and with portions resembling soft carcinoma ; the gall-bladder was enveloped in this diseased mass, but was merely softened and had its internal surface tumefied into nipple-shaped excrescences. From the midst of this softened mass, two ulcerated openings led into the descending portion of the duodenum and the transverse arch of the colon—adhesions prevented any communication with the peritoneal cavity. In the vicinity of the ulcerated openings the mucous membrane of the intestines was diseased; with respect to the duodenum the disease occupied its first and second portions and a part of the third, which were of their usual calibre; but the remainder of the small intestines were free from disease and much contracted in their diameter. The disease of the colon extended both ways, backwards to the cæcum and onwards throughout its whole length and even into the rectum. This disease consisted chiefly of a dark ecchymosed appearance of the valvulæ conniventes of the duodenum and the longitudinal plicæ of the colon, with thickening of the submucous tissue. The mucous membrane of the stomach was not obviously diseased, but the change in its appearance from the pylorus to the duodenum, where the opening existed from the softened tumor, was very abrupt.

*Chest.* The only morbid appearance discovered in the chest was an atheromatous, and in one spot earthy deposit at the commencement of the aorta, and an interstitial pliant deposit in the mitral valves.

Case 5. *Long continued ague with chronic diarrhœa—enlargement and softening of the liver with biliary calculi impacted in the gall-bladder—the products of peritoneal inflammation of the surface of the liver and intestines—internal surface of the stomach coated with tenacious layer of mucus—morbid appearances denoting chronic colitis, with ulcerations in the process of healing.*

E. M. æt. 36, widow and plat-worker, was admitted Nov. 2, 1837. She had had various attacks of ague ever since the preceding March—the type was at first quartan, but had become quotidian. During four months preceding her admission she repeatedly had attacks of diarrhœa, and, for two months, had suffered from pain in *left* hypochondrium, increased after each paroxysm of ague. At the period of her admission she was still labouring under ague, profuse diarrhœa and pain, with tenderness of the left hypochondrium; and presented a wan, sallowish aspect—appetite and digestion were at that time not greatly impaired. A blister was applied to the left hypochondrium and a few grains of sulphate of quinine directed to be sprinkled over the blistered surface—at the same time, a combination of hyd. c̄ cret. and pulv. ipec. comp. and liquor calcis, were used to combat the diarrhœa. Under this treatment, the ague ceased and the diarrhœa was checked; but, on the 9th, a large but superficial slough had formed on the blistered surface, and she complained of pain with tenderness at the epigastrium, and had a very anxious look. The slough separated in a few days, and left an extensive granulating and suppurating surface; by this time (9th) the tongue had become very dry in the centre, and viscid and red, and the epigastric pain continued. On the 14th the diarrhœa again returned; from this time to the 16th of the following month, she had frequent relapses of diarrhœa, for which various remedies were used for the most part ineffectually, with the exception of the sulphate of copper, which given in ¼ of a grain doses every four or six hours had a considerable effect in controlling this state of the bowels. During this time her general condition varied very considerably, the appetite sometimes being good and the stomach capable even of digesting animal food in small quantities without aggravating the symptoms; while at other times food of any kind could scarcely be tolerated, and the pain in the epigastrium and left hypochondrium, with a very red tongue, returned.

On the 16th of Dec. the tongue again became dry and glabrous, and the abdomen was tense and resonant, with pain and tenderness in the *right* hypochondrium and right lumbar region. From this period the most urgent symptoms continued to be referrible to this region of the abdomen, with a relapse of pain in the epigastrium, and afterwards a return of profuse diarrhœa. This state became gradually worse, till these symptoms, with the exception of the diarrhœa, were exchanged about the 6th of January, for those of sinking and collapse, which terminated in death on the 18th; during this period (from the 16th Dec.) little relief was derived from any remedies, a suppository of opium alone mitigating the diarrhœa—at an earlier period a few leeches had relieved the abdominal pain.

*Post-mortem examination, nine hours after death.*—Great emaciation—œdema confined to the right lower extremity. The liver enlarged and reaching as low as the umbilicus, was covered with patches of soft adventitious membrane; the structure of the liver was soft, friable, and pale. The gall-bladder was contracted and elongated, and entirely filled by seven or eight gall-stones of the size of small marbles, sacculating the bladder. The small intestines, extremely dark, were glued together by cellular adhesions: from one to two pints of fluid were contained in the peritoneum. The spleen was of natural size and consistence, presenting only on its surface, slight, thin and white patches. The mucous membrane of the stomach was besmeared with a large quantity of mucus, as were the valvulæ conniventes with mucus tinged with bile. The cæcum, ascending colon, and transverse arch presented a healthy mucous surface: there was however an asc. lumbric. in the cæcum; and the arch was much dilated. The descending colon and rectum were greatly diseased—the coats being much thickened; the change from healthy to unhealthy mucous surface was quite abrupt, presenting a clean line of separation. The internal surface presented a dark, thickened, uneven condition of the membrane, with insulated fungiform portions; the whole patch which occupied the entire calibre of the descending colon, sigmoid flexure, and the greater part of the rectum, had the appearance of ulceration in the process of healing, being below the level of the adjoining healthy mucous surface. The large intestines contained a quantity of solid fæces. The neck of the uterus was of cartilaginous hardness.

---

### ON THE BEST MEANS OF APPLYING PRESSURE TO THE UTERUS AFTER DELIVERY.

*To the Editors of the Medical and Chirurgical Review.*

GENTLEMEN,—The important principle of making such pressure on the uterus as will ensure its contraction immediately after the birth of the child, in every case of labour, is at length so fully established as to be received throughout the profession as one of the axioms of scientific midwifery. Every practitioner carries out this principle by some one or other of various methods which suggests itself to his mind,—from the simple pinning of the long napkin to the formidable tourniquet and rolled pillow. With the ulterior desire of eliciting further mechanical improvement, I proceed to give an explicit description of two bandages which I have long used in my practice; and some medical friends, whose judgments I highly value, have tried them with the greatest satisfaction.

In forming these bandages my first object was to make pressure on the region of the uterus by a *firm unyielding substance;* because, by this means, the contraction of that organ was found not only to be more readily produced than by a similar degree of force applied by means of a bandage composed of linen or of any soft substance, but, having been so produced, was more readily maintained. To this principle we must refer the signal benefit derived from pressure by the firmness of the hands in cases of sluggish uterus; but hands and arms soon tire at this employment, and consequently the degree of pressure necessary to the complete contraction of this organ, instead of being steadily continued, becomes relaxed, and hæmorrhage occurs; or if it has been momentarily suspended, is renewed.

Figure 2, in the subjoined engraving, represents a piece of mill-board, obtained from the stationer, seven inches by eight, padded on the inside with two layers of wadding, and covered with flannel or keth flannel or kerseymere. This plate has been previously divided down the middle, as seen in fig. 3; then united by pasting a strip of leather on each side, so as to form a joint; thus enabling it to be folded into half its compass, like a closed book; and, with the band, fig. 1, wrapped round it, to be conveniently put into the pocket. The band, fig. 1, which is made of variable length to suit the different dimensions of different females, is composed of webbing, three inches wide; is furnished with two buckles, and three sets of straps to regulate its pressure; and has four inches of India-rubber web let into it, so as to combine a degree of elasticity with the force of its pressure. Fig. 3 shews the bandage duly applied; the band being under the crests of the ilia, and carried round the hollow of the back, just at the junction of the sacrum with the spinal column, by which it is prevented slipping upwards. This bandage, from its easy application, I use immediately after the birth of the child, directing the nurse, if there be hæmorrhage, to increase the pressure by buckling it tighter. This simple bandage answers well for every purpose proposed, is capable of exerting a great degree of pressure, and of thus facilitating or accelerating the complete contraction of the uterus.

When the patient is comfortably in bed, I usually apply what I call my sash bandage: were it applied previously it would probably become soiled. This is represented, fig. 7, applied under the crests of the ilia, and carried to the hollow of the back, just above the sacrum. Fig. 5 represents the exterior, and fig. 6 the interior of exactly the same plate, with the joint as described in fig. 2; but on each side, within two inches of the bottom, are two holes through which a piece of tape is seen passed from the inside, to attach a pearl button on the outside of the size of half-a-crown, as seen in fig. 4. Fig. 4 shews this bandage before it is applied, folded in half: it is about 30 inches long, and made of white jean doubled; it incloses the plate, fig. 2; it tapers from the width of the plate towards each end, where twelve inches of strong broad tape are attached for tying under the buttons, as seen in fig. 7.

The bandage is sloped downwards to fit the hollow above the sacrum, and in its posterior portion a slit is made, through which its opposite end is passed. By placing the plate over the region of the uterus, carrying the two ends of the bandage to the hollow above the sacrum, and then bringing them round under the crests of the ilia—drawing them tightly over the plate, and tying the tapes in a firm manner under the buttons—a very effectual resistance is offered to the tendency which otherwise every bandage would have to slip upwards, and recede from the part which should receive pressure.

The specific advantage of the above plan, besides affording an extraordinary degree of comfort to the patient by the support it affords to the relaxed abdominal parietes, thus preserving the natural figure, is found by experience to be the prevention of uterine hæmorrhage and its dreadful consequences. Under the pressure which this bandage is capable of producing, even the formation of a coagulum of any size is almost impossible, and thus the accoucheur is enabled to leave his patient in a state of perfect security, which never can be the case if the uterus, though contracted at the time, be left without the support of some such pressure, which is therefore essential in every case in a greater or less degree. The natural expulsion of the placenta will be much accelerated by systematic pressure. Dr. Ruysch first, and afterwards Drs. Denman and Wm. Hunter, vainly imagined that the musculus orbicularis Ruyschii was self-sufficient for the expulsion of the placenta, and also for the complete contraction of the uterus; but even this muscular power is very greatly increased by the aid of pressure. I could give the detail of several cases of retention of the placenta, which I have recently seen in consultation, where the uterus sympathizing with the general system, after a protracted labour, was in an atonic state. In these cases, as there was no hæmorrhage, I advised the continued permanent pressure by means of my bandage. The inherent muscular power of the uterus, thus assisted, safely expelled the placenta, in every one of these cases, without the introduction of the hand, and without any hæmorrhage. In one or two of these cases the placenta was retained three days and nights without any untoward symptom, and then securely and satisfactorily expelled by the natural efforts. A manifest mitigation of after-pains is acknowledged to be produced by means of these bandages by many females who have previously borne children, and have had no such assistance.

By securing the permanent contraction of the uterus, many cases of puerperal fever may be prevented, for it may be confidently asserted that puerperal fever is frequently produced by a congested state of the uterus. Nine tenths of the diseases of the uterus, especially the chronic and acute engorgements, may be traced to the condition of that organ after parturition, and may be prevented by means of a proper pressure, such as the bandage described is calculated to make.

We have much reason to wish that those who have witnessed the accurate precision with which the various diseases of the uterus are discriminated and treated at the different institutions at Paris, where I am given to understand the most rapid progress has lately been made in this department of our art, would throw some light on this obscure subject. We might then be led to abandon the absurd system of fighting with the symptoms of the disease of the uterus, as though they were in themselves diseases, for the adoption of a rational system founded on accurate knowledge of the various alterations of structure which produce those symptoms.—Your obedient servant,

15, *King's Row, Pentonville,* J. L. Fenner.
*June* 11, 1838.

---

"A Crust for the Critics."

*To Dr. Forbes and Dr. Conolly.*

Gentlemen,—Incessant professional occupation has hitherto prevented me from replying to your illiberal and malicious attack, in your so-called review on my work, The Philosophy of Marriage, which appeared in one of the late numbers of your periodical. For my own part, I should have deemed your criticism totally unworthy of notice, as it is well known by all in the least acquainted with the periodical literature of the medical press in this country, that there was something like an old score, or *quid pro quo,* on your part, to be settled between us, after my former just animadversions upon your pitiful productions. This is now evident from the fact that you, and you alone, of all the medical reviewers, my former rivals, thought it a convenient opportunity to attack my work as soon as I had ceased to be a rival editor, for you durst not have done so before with impunity, as you are very well aware.

It also appears that you have not the common candour of allowing those whom you attack an opportunity of defending their works in your pages, and much less the space, unless by paying heavily for the mere insertion of retributive and just defence; notwithstanding that journals which have, and will continue to have, a far greater circulation than yours, invariably admit the insertion of replies to their criticisms. Availing myself of this liberal privilege afforded by the *Medico-Chirurgical Review,* which, by the way, has a circulation your *precious* Review can never even approximate, in consequence of the injudicious manner in which it was begun, and has been hitherto conducted, I now reply to you.

Had you not started aside from the usual observance of etiquette amongst fair and honorable editors, you would not, as I above intimated, have unwarrantably and maliciously attacked a former editor, solely because he conscientiously censured your productions, on account of their want of any, but negative merit.

When you published the first part of the Cyclopædia of Practical Medicine, I felt it my duty to criticise it in the spirit of truth and science; and pronounced it a meagre and spiritless compilation, far below mediocrity, made up, for the most part, of extracts from the old writers, without any useful additions derived from the then actual advanced state of science.

The contributors were, in general, unknown writers, mere literary adventurers, the majority of whom were not versed in medical literature, and evinced but little skill in composition.

These strictures, which were certainly merited, gave you deadly offence. Some more accommodating journalists, it is true, lauded the work at first to the skies, merely to gratify you and the publishers; but even these candid and honest critics were eventually compelled to approve of the judgment I had passed upon it. I again maintain, that no well-informed physician in the kingdom will refuse to admit, that many of the articles are badly done, that the work might be reduced to half the size and price, which by the way was the determination of the late Mr. Sherwood, if ever a future edition was required; and that, as a whole, it is far inferior, indeed can scarcely be compared, with the truly learned, practical, and unequalled Dictionary of Medicine, by Dr. Copland. In fact, all acquainted with the French Medical Dictionaries, are disgusted with your production. It is too long-winded—or, to use a French proverb, Ouvrage de longue haleine. Nevertheless, you are, as part owners of the work, incessantly puffing it in your journal: a most disinterested proceeding, truly, on your part, while you are as incessantly either abusing or sneering at the works of able and more judicious writers.

The next ground of offence was announcing it as my conviction, that your Quarterly Journal was, in merit and usefulness, far inferior to the *Medico-Chirurgical Review*, and could never approach it in circulation, in the way it was conducted.

I now ask you, has not my judgment been fully verified? Is the sale of your unfair and heavy periodical, one-half of that of the Medico-Chirurgical Review? According to the Spanish proverb, after having cried up your wine, you sell vinegar—Aviendo pregonado vino, vend vinagré.

Non omnia possumus omnes.

I shall not here advert to my comments upon your professional pretensions to high practice, and certain official situations in this metropolis; they have been duly appreciated by the public, and you are still left in the provinces to enlighten and abuse medical authors in general.

Under all the preceding circumstances, it cannot be very surprising to the reader, that you should abuse my productions, so soon as I ceased to be a journalist, and no longer possessed the means of reply in my own power.

Had you confined yourselves within the limits of fair and impartial criticism of any of my humble works, I should never have complained; but when you wilfully and wantonly misrepresent them, extract parts of sentences, so as to destroy the context, misquote and substitute whole sentences and expressions for others, which you well knew were too ignorant and absurd to be written by me, or by any educated member of the medical profession, I have every right to complain, and to expose your unfair and dishonest mode of criticism, to the contempt and derision of the medical profession in all countries.

Now for examples of your criticism in proof of the preceding charges—

In your review of my edition of Dr. Denman's Obstetrician's Vademecum—you quote the following sentences which are not mine, nor are they in my edition of that work. "Come under the *neck* of the pubes." No. 4. p. 524. I never used such a barbarism as *neck of the pubes*. I do not know such a part of the human body as the *neck* of the pubes, though your *placentæ prævia reviewers may*; and I consider that ascribing the use of such a term to me was a malicious insult.

Again, you state—"The ergot of rye," Dr. Ryan says, "will 'always' effect the removal of a retained placenta. This is surely attributing more certain powers to the ergot than it really possesses." I never wrote or said any such nonsensical sentence; and it is scandalously introduced, to enable the stupid and ignorant reviewer to knock down "a windmill of *his own* erection."

Again—" with respect to the 'half ounce' doses of the ergot—we never have heard the medicine was exhibited in such quantity," p. 525. My words are "The maximum dose is ʒiss." p. 68. This is another windmill, the erection of a stult, on whose shallow capacity I have previously commented. This I now repeat, as well as my former opinion, that this worthy betrays a gross ignorance of practical obstetricy from the beginning to the end of his review of the Obstetrician's Vademecum. He has, lastly, done me the additional favour of once more misquoting me—" to *breaking down the os uteri* with the finger."

Et sic de similibus et de cæteris.

Was there ever such misrepresentation among gentlemen of a liberal profession?

*Breaking down the os uteri!!!*

Do you call this fair and honest reviewing? I shall leave the profession to judge.

With reference to your review of my work on Marriage, it is not a review, but a virulent attack, and such I am proud to find is the general opinion of those few of the profession who have perused it—men infinitely your superiors in capacity and attainments. You designedly passed over the preface and introductory remarks, which proved the important object of the work, in as much as theologians, philosophers, physiologists, legislators, and lawyers, as well as the medical profession of all ages in civilized countries, have fully discussed every question relating to the reproductive functions, as well as the laws relating to marriage, bastardy, divorce, seduction, infanticides, homicides, and numerous other crimes, upon which an infinity of questions arise, deeply interesting to every class of society. But to the point. It would seem that you are apt to be intentionally blind or incapable of comprehending the various bearings of the numerous important questions that daily occur concerning abuses of the functions just mentioned, in relation to life, liberty, honour, property, &c. &c.

Damnant quod non intelligunt.

It did not suit your purpose to dwell duly upon the prefatory matter, for it would have totally upset your superficial remarks, and your unjustifiable charge that the work is immoral—though you knew it was the reverse.

According to your sapient dogmas, all works on anatomy, midwifery, law, medicine, morality, political economy, the public press, and even the Bible itself, ought to be suppressed. Now this may be the code of "the little great men of Chichester and Worcester," who fancy they can lay down laws as well as circumscribe the limits of imparting knowledge, but certainly not for men who think for themselves. Your code is unfortunately at variance with the universally-received doctrine at this period of the nineteenth century, namely, the importance of "the diffusion of all useful knowledge amongst mankind." Never did the medical profession or public condemn the diffusion of natural science. I can readily imagine your sardonic grins when I inform you, that notwithstanding your unwarrantable censure, my work has met with the approbation of many of our sound philanthropic medical philosophers, and other distinguished personages, including clergymen of every denomination, with whom you can have no pretensions. A large impression, 1500 copies, sold in one year, and I am now, notwithstanding your criticism and condemnation, about to publish a new and much larger edition. How true the French proverb—Il y a des reproches qui louent, et des louanges qui medisent. Some reproaches are a commendation, and some praises detraction. Laudatur ab his, culpatur ab illis. The sure way to be deceived is to believe ourselves more cunning than the rest of the world—to abuse every one with great eloquence and little conscience, or as the Italians render it—" Di grand eloquenza, picciola conscienza"—but we should remember, that curs that are always barking generally get sore ears. Les chiens hargueux ont toujours les oreilles dechirées.

I hope I may be allowed to observe in this place, without much vanity, that there are few who have studied medicine in all its branches, both in this kingdom and abroad, with more zeal and industry than I have, and that few have evinced more researches in so many different original works. In fact, these have been more than favourably noticed both at home and abroad; while a few hireling pseudo-critics at home, who, like the Swiss amanuenses, readily undertake "what is above or below their capacity," gravely declared that those very works *were the worst ever published.* Nevertheless I must take leave to observe, that not one of these truly impartial and erudite critics has produced a single original work of the slightest value, while most of mine have passed through several editions, both in this and other countries. I mention these facts to shew the high claims of my worthy assailants, either as learned authors or qualified medical critics; most of whom have not had sufficient talents or attainments to produce an original sixpenny pamphlet; while the few of them, who have ventured to appear as authors, and all of them as quondam rival journalists, can never forget or forgive my just strictures and censures upon their paltry productions, and dishonest periodicals. *Hinc illæ lachrymæ.* Hence, reader, the cause of their vituperative attacks.

I now allude to these facts to shew how little I care for unfair or unprincipled medical critics; and also to prove, that their censures have not, in any way, prevented repeated editions of the very works of mine, which they so loudly and unjustly abused and condemned. I may likewise be permitted to add, that my practice far exceeds that of my spiteful and mendacious assailants. Such are the bad effects of unfair and unmerited criticism. As a further proof of the truth and force of these remarks, I have to observe, that there are no three medical editors in this kingdom who have been so unsparingly abused as Dr. James Johnson, Dr. Copland, and myself. Indeed it is my own firm conviction, that partial and malicious criticism has never done myself, nor any other individual, any real injury.

I can aver, after careful and extensive observation and experience, that a more unprincipled and incompetent set of medical critics, with a few honorable exceptions, do not exist than in this country. Did not our infamous, sophisticated, and unchristian libel law, which enacts, "the greater the truth the greater the libel," restrain me, I could enter into details which would fully prove the truth of the preceding strictures.

Suffice it then, to observe, that many of our medical critics allow private feeling, party prejudice, self-interest, and a variety of other bad motives, to influence them in their reviews. They pass over or abuse valuable works, while at the same time, they praise miserable productions, which speedily find their way to the butter-man, the trunk-maker, and "serve to put under pies, to lap spice in, and keep roast meat from burning—quos legunt cacantes."

How often had I, whilst a critic, justly praised really valuable works, which were afterwards censured in the severest terms by most of my contemporaries; and how often had I as justly censured other productions, which they lauded "to the fifth heavens."

It is very painful to me to admit, but it is the truth, that our medical reviewers in general want the talent, erudition, candour, honesty, and impartiality of their contemporaries in France, Germany, Italy, and other European nations, as well as in America and India. Every one knows that there are the most able medical writers and critics in this country, but the latter are very unfortunately the smallest portion.

But to return from this digression, I have to observe, that it is really lamentable to see critics, of your calibre, finding fault with all authors for want of original matter. It would be very important to the medical world, were you to inform it, where we can find original matter. Most assuredly not in your Cyclopædia or Review, though you who aim at tomes, the offspring of other men's brains, condemn works by wholesale; in fact, you attack every thing unless the production of a friend, for want of originality. The laborious and learned productions of Professor Cooper, Dr. Copland, Dr. Beck, as well as my own humble works, fall under the ban of your erudite critical censure. Pray what original work have you produced? If any, when did it appear, and where is it to be found? At any rate, I have never seen one scrap of original matter upon any medical or other subject from your leaden pens.

Ever since one of you published his Homer, that is to say, his translation of Laennec's work, which by the way, a medical tyro of two years standing could as well accomplish, you have given no proficiency in medical literature, to say nothing of science, so far as I have observed, and I am not aware, that the science or practice of medicine is indebted to either of you "par nobile fratrum," for one single fact, in relation to its advancement.

How well qualified must you then be to decry mine or any other work, for its want of originality, while you yourselves, have evinced none!

Admitting for the sake of argument, what is not true, that there is not "a syllable of original matter in the work" (mine); in such case you and I are precisely in the same position. So much for your love of originality.

But permit me to inform you, that your assertion, is what our polite Gallican contemporaries would designate, cela n'est pas vrai.—Anglice—not true.

No sabe uno que pensar de vm.

One does not know what to think of you. You know, or you ought to have known, before you made the preceding remark—had you condescended to peruse my work—that more than one half of it is original matter;—a fact, which ill accords with your stricture—"there is not an original syllable in the work."—p. 460. Such is your remarkably impartial criticism.

You in common with too many medical writers of the day, attack compilers of works, as if every medical book was to be original. This illiberal and foolish observation was made on a former occasion, by a brother critic of yours' on my work on Medical Jurisprudence, whose *interest* it was to praise the learned and elaborate compilation of Professor Beck, as it was then published by his employers, against the new edition of mine, which was declared on that occasion, the worst book ever published by this honest reviewer, though lauded in America, which has much reason to be proud of Dr. Beck—where the reviewers declared it the best manual extant—not a systematic treatise—that it ought to be reprinted in that country, which was accordingly done, under the able editorship of Professor Griffiths.

Now hear the sentiment of a first rate moral philosopher, poet, and critic, of the Augustan age of classical literature in this country.

"Were all books reduced to their quintescence, many a bulky author would make his appearance in a penny paper: there would be scarcely any such thing in Nature as a folio: the works of an age would be contained on a few shelves; not to mention the millions of volumes that would be utterly annihilated."—*Addison.—Spectator, No.* 124. Your Cyclopædia and Journal excepted!

It would be well, were many modern medical critics to remember, that a compiler is one deeply versed in all that has been written on his subject; not one of those pretended original authors, who is ignorant of the labours of his predecessors, and who sets forth his original opinions, which were really published centuries before he was born, as is too generally the case.

Lastly, you attack my article on Abortion in the Cyclopædia of Practical Surgery. I shall not now state your position with respect to that work; but I shall inform you that my article is not as you allege, copied, from my learned and able friend Dr. Copland, but was expressly written by myself, and afterwards sadly modified in the work, in which it appeared. Many of our cyclopædists are like the Swiss Amanuenses already noticed; but I must beg to assure you, that the article, such as it even now is, with all due deference to your obstetric opinion, is not below the present state of science, for it contains many practical hints, which I defy you to point out in any other work extant.

Allow me to observe, in conclusion, that when you condescend to review any of my unfortunate works in future, pray, for your own sakes, employ some persons, who know something of the subject matter. Vaya vm. con Dios hasta la vista—Good-bye, till we meet again.

I am, Gentlemen,

Your obedient Servant,

4, Charlotte Street, Bloomsbury, Bedford Square, M. RYAN, M.D.

December 12, 1838.

---

*To the Editors of the Medico-Chirurgical Review.*

GENTLEMEN.—In your last Number, you have done me the honor to notice a Paper of mine on Prolapsus Uteri, read before the Medical Society of this State at its last session. You will pardon the liberty I take in calling your attention to a very strange error under which the writer of the notice appears to have penned his remarks. I am not the inventor of the apparatus called "Supporter" nor of any improvement therein, neither do I reside at 279, Regent-street, London! The apparatus was invented by the late A. G. HULL, M.D. of this city, seven or eight years since, and has not, so far as I am aware, undergone any modification since its introduction into practice in this country.

The Medical Society in which I read the Paper is a representative body, consisting of one medical man from each county in the State, elected by the medical society of such county to act as its delegate, and holding annual sessions, at the seat of government. This society is established by charter, and forms a part of our sanatory police. At its meetings the delegates read papers, which if approved by the society, are on motion referred to an editorial committee for publication in the "Transactions."

Being delegate from the County of New York, where the new apparatus was in extensive and successful use, and having applied it, in many instances successfully in my own practice, I wrote the Paper you found in the last Number of the Transactions, and were so kind as to notice, as a species of local intelligence from my constituents. The Paper is entitled (I quote from memory) "*Observations on Prolapsus of the Womb, with reference to the Modus Operandi of a New Apparatus, invented by the late Dr. Hull, called Utero-Abdominal Supporter,*" or words to this effect.

If you will take the trouble to look at your copy of the Transactions again, and make a brief correction of the errors, you will very much oblige me.

I shall call to-morrow on the Messrs. Woods, to ascertain if they will consider themselves at liberty to correct the American edition, which is probably now in press.

Very respectfully, your obedient servant,

JOHN F. GRAY.

*New York, Nov.* 21, 1838.

## Figures and Tables

**Figure f1:**